# Repurposing clinically available drugs and therapies for pathogenic targets to combat SARS‐CoV‐2

**DOI:** 10.1002/mco2.254

**Published:** 2023-05-14

**Authors:** Yiying Xue, Husheng Mei, Yisa Chen, James D. Griffin, Qingsong Liu, Ellen Weisberg, Jing Yang

**Affiliations:** ^1^ Department of Hematology, Tongji Hospital, Frontier Science Center for Stem Cell Research, Shanghai Key Laboratory of Signaling and Disease Research, School of Life Sciences and Technology Tongji University Shanghai China; ^2^ Anhui Province Key Laboratory of Medical Physics and Technology, Institute of Health and Medical Technology, Hefei Institutes of Physical Science Chinese Academy of Sciences Hefei China; ^3^ University of Science and Technology of China Hefei Anhui China; ^4^ Hefei Cancer Hospital Chinese Academy of Sciences Hefei China; ^5^ Department of Medical Oncology, Dana‐Farber Cancer Institute Boston Massachusetts USA; ^6^ Department of Medicine, Harvard Medical School Boston Massachusetts USA

**Keywords:** combination therapy, drug resistance, pathogenic targets, repurposing therapies, SARS‐CoV‐2

## Abstract

The coronavirus disease 2019 (COVID‐19) pandemic has affected a large portion of the global population, both physically and mentally. Current evidence suggests that the rapidly evolving coronavirus subvariants risk rendering vaccines and antibodies ineffective due to their potential to evade existing immunity, with enhanced transmission activity and higher reinfection rates that could lead to new outbreaks across the globe. The goal of viral management is to disrupt the viral life cycle as well as to relieve severe symptoms such as lung damage, cytokine storm, and organ failure. In the fight against viruses, the combination of viral genome sequencing, elucidation of the structure of viral proteins, and identifying proteins that are highly conserved across multiple coronaviruses has revealed many potential molecular targets. In addition, the time‐ and cost‐effective repurposing of preexisting antiviral drugs or approved/clinical drugs for these targets offers considerable clinical advantages for COVID‐19 patients. This review provides a comprehensive overview of various identified pathogenic targets and pathways as well as corresponding repurposed approved/clinical drugs and their potential against COVID‐19. These findings provide new insight into the discovery of novel therapeutic strategies that could be applied to the control of disease symptoms emanating from evolving SARS‐CoV‐2 variants.

## Graphical abstract

In this review, authors provide a comprehensive overview of various identified pathogenic targets and pathways, as well as corresponding repurposed approved/clinical drugs and their potential against COVID‐19. Moreover, authors summarizenumerous potential pathogenic targets associated with viral life cycle and virus–host cell communication, repurposed drugs and their mechanism‐of‐actions for pathogenic targets that have been vetted for safety, and repurposed drugs that are approved or clinical available for COVID‐19.And thenthe future prospects of drug repurposing strategies in discovery and development of pathogenic targets and novel medicines as well as effective therapeutics for combating COVID‐19 and other infectious diseases that are current or on the horizon are discussed.

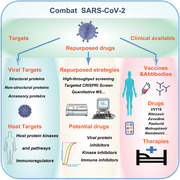

## 1 INTRODUCTION

Severe acute respiratory syndrome coronavirus 2 (SARS‐CoV‐2) caused an outbreak that started around December 2019 and quickly spread.^1^ The outbreak caused a global threat to human physical and mental health worldwide.^2^ According to Centers for Disease Control and Prevention, the effects of coronavirus disease 2019 (COVID‐19) may be long lasting.^3^, ^4^ The pandemic nature and continuous evolution of the virus produced additional, novel immunity‐dodging coronavirus variants of concern, including B.1.1.7 (Alpha), B.1.351 (Beta), P.1 (Gamma), B.1.617.2 (Delta), B.1.1.529 (Omicron), BA.4, and BA.5, with stronger transmission ability and more immune evading potential, leading to reinfections and breakthrough infections across the globe (COVID‐19 Dashboard). The COVID‐19 disease is characterized by pneumonia that progresses to dyspnea, acute respiratory distress syndrome (ARDS), and multiple organ dysfunction syndrome.^5^, ^6^ According to the World Health Organization Dashboard, SARS‐CoV‐2 has caused more than 6 million deaths up to now, with confirmed cases over 600 million.^7^ Moreover, some experts now expect it to become endemic, similar to the common cold and the seasonal flu that appear year after year (NBC news, Europe Newsletter). Although the advent of vaccines and antibodies abated the global burden, they showed variable effectiveness against the new variants of SARS‐CoV‐2 due to the structural proteins of coronaviruses (i.e., Spike protein) undergoing rapid mutations due to high selective pressure. In addition, the fact that certain immunosuppressed individuals are unable to mount responses even after two vaccine doses suggests a need for other options to protect against SARS‐CoV‐2 variants.^8^, ^9^ Therefore, efforts have been made to search for and identify proteins that are highly conserved across multiple coronaviruses that could serve as potential therapeutic targets for COVID‐19 treatment.^10^ In addition to viral potential targets of coronaviruses, such as the replication‐related enzymes RNA‐dependent RNA polymerase (RdRp), protease Mpro/3Cpro, and PLpro,^11^ there are many other attractive targets that play roles in the virus–host response. One example is the protein kinase AXL, which acts as a virus–host cell receptor.^12^, ^13^ Currently, researchers are attempting to identify treatments that suppress the transmission of SARSCoV2 or ameliorate the symptoms of COVID19, and this trend warrants the development and repurposing of approved agents blocking crucial targets integral to viral infection and host response.

The cost‐effective repurposed approaches have been investigated against key host cell targets of SARS‐CoV‐2 for potential to contain the spread of existing SARS‐CoV‐2 variants as well as to counter future variants.^14^ An efficient and economical approach is to identify new therapies for diseases, particularly in those cases where there is an urgent need for new therapeutics and preclinical safety studies have already been conducted. The advent of computational approaches and genetic programming has led to the development of novel strategies for drug repurposing, which offer a convenient alternative in the advent of an unexpected medical crisis, such as the outbreak of COVID‐19.^15^ Several oral antiviral drugs for COVID‐19 have been successfully repurposed and on the market with promising therapeutic effects. Paxlovid is a SARS‐CoV‐2 protease inhibitor antiviral therapy developed by Pfizer Inc., which reduced the risk of hospitalization or death by 89%.^16^ Paxlovid is comprised of nirmatrelvir and ritonavir. Nirmatrelvir prevents the virus from growing and spreading, and ritonavir prevents nirmatrelvir from being metabolized in the body long enough to exert its effects. Molnupiravir, developed by Ridgeback Biotherapeutics and Merck Sharp & Dohme (MSD), is an antiviral medication used to treat COVID‐19. Molnupiravir was investigated in a clinical trial and reduced the risk of hospitalization or death at‐risk with COVID‐19 (NCT04575597). Azvudine is a small molecule oral drug developed by Real Biotechnology Co., Ltd that selectively inhibits the activity of SARS‐CoV‐2 RdRp, and therefore inhibits virus replication.^17^ The clinical trial (NCT05033145) showed that azvudine treatment accelerates the elimination of virus and reduces mortality. VV116 is another new oral nucleoside antiviral drug, developed by Junshi Bio‐Pharmaceutical Technology Co., Ltd that was demonstrated in three clinical trials to lead to shorter recovery time for patients as compared with Paxlovid (4 days vs. 5 days) (NCT05227768, NCT05201690, NCT05221138).^18^ Findings reported with these agents suggest that drug repurposing is an effective way to treat disease outbreaks such as COVID‐19.

This review will include some of our recent, published studies and will include four parts: (1) Summary and characterization of numerous potential pathogenic targets associated with viral life cycle and symptoms of infection; (2) summary of repurposed, approved/clinical drugs for pathogenic targets that have been vetted for safety and that are more accessible for COVID‐19 treatment, including mechanism‐of‐action; (3) summary of repurposed drugs that are approved or in clinical trials for COVID‐19; and (4) discussion and future prospects, aiming to provide valuable insights into the discovery and development of novel medicines as well as effective therapeutics for combating COVID‐19.

## 2 POTENTIAL PATHOGENIC TARGETS OF SARS‐CoV‐2 FOR ANTIVIRAL THERAPEUTICS

**FIGURE 1:**
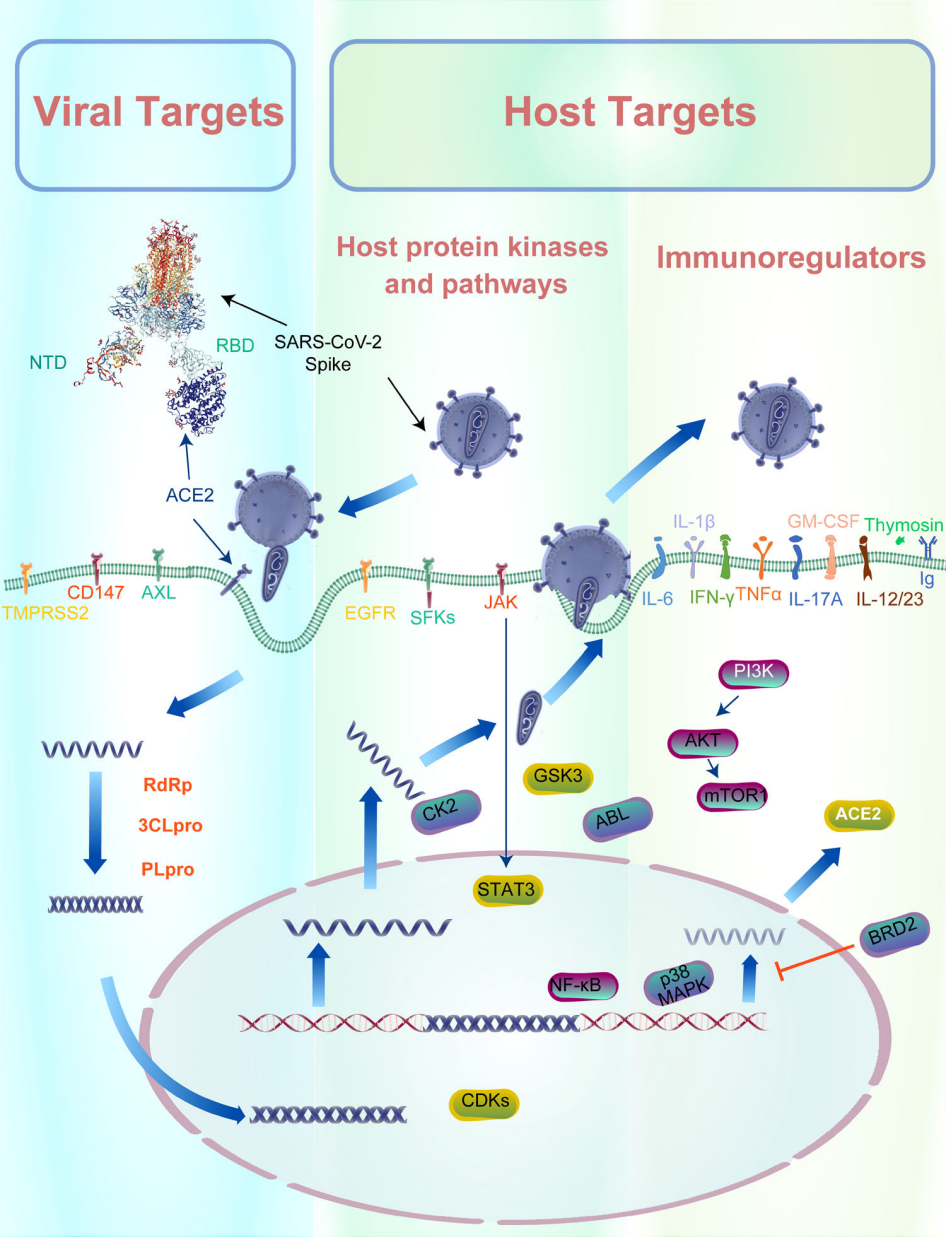
Potential pathogenic targets that are essential to SARS‐CoV‐2 viral life cycle and virus–host cell response. Two main categories of potential therapeutic targets that are involved: (1) direct viral targets and (2) host cell protein kinases, signaling pathway, and immunoregulators targets. The life cycle of coronaviruses begins when the virion binds to the host cell receptor (angiotensin‐converting enzyme 2 (ACE2), the cell surface serine protease TMPRSS2, and tyrosine‐protein kinase receptor UFO (AXL) kinase and host cell receptor CD147) via its Spike protein S, which promotes viral uptake and fusion at the cellular or endosomal membrane. The serine protease, Transmembrane Serine Protease 2 (TMPRSS2), is critical for Spike protein priming and facilitating the fusion of the viral and host membranes. Nonstructural proteins as antiviral targets for SARS‐CoV‐2: NSP12 (RdRp), which enables viral genome replication, NSP5 main protease (MPro) or 3‐chymotrypsin‐like protease (3CLpro), and NSP3 papain‐like protease (PLpro), which are essential for the viral infection cycle and replication. The cellular kinase proteins and pathways in particular appear to be essential for the life cycle of the virus. Kinases, such as Abelson tyrosine kinase (ABL), the SRC family of kinases (SFKs), AXL kinase, the Numb‐associated kinase (NAK), the tyrosine kinase receptor epidermal growth factor receptor (EGFR), PI3K/Akt/mTOR pathway signaling mediators, cyclin‐dependent kinases (CDKs), casein kinase 2 (CK2), p38 MAPK, JAK–STAT, and GSK‐3, are involved in transmission, pneumonia‐like symptoms, inflammation and fibrosis associated with SARS‐CoV‐2 infection. Targeting these factors are currently a significant effort on the part of numerous pharmaceutical companies. The complex immunopathological manifestations of COVID‐19 are associated with many signaling pathways, including JAK/STAT signaling, NF‐κB, MAPK pathways, which lead to an overwhelming release of inflammatory mediators, such as tumor necrosis factor (TNF)‐α, interferon (IFN)‐γ, GM‐CSF, interleukin (IL)‐1β, IL‐6, IL‐12/23, IL‐17A, thymosin and immunoglobulin (Ig). This potentially causes a life‐threatening cytokine storm associated with extensive lung injury in SARS‐CoV‐2‐infected patients.

In order to develop potential antiviral medicines to constrain this global pandemic, researchers are working to understand the underlying pathology of COVID‐19 and identify the druggable molecular targets for total containment of the disease.^10^, ^19^ Some targets are proteins or pathways that are highly conserved among multiple viruses, such as Ebola virus, MERS‐CoV, and more recently, coronaviruses.^11^, ^12^, ^13^ There are two main categories of potential therapeutic targets that are involved in SARS‐CoV‐2 infection and symptoms: (1) the viral targets associated with SARS‐CoV‐2, (2) the host targets (host protein kinases, pathways, and immunoregulators) essential for viral life or virus–host cell response^14^, ^20^, ^21^, ^22^, ^23^, ^24^ (Figure 1 and Table 1).

**TABLE 1:** Summary and characterization of numerous potential pathogenic targets associated with viral life cycle and virus–host cell communication.

Categories		Pathogenic targets	Pathogenesis relevance	Mechanisms of action	References
Potential therapeutic viral targets associated with SARS‐CoV‐2		Spike glycoprotein	Plays a critical role in the initial steps of pathogenesis	Through binding to ACE2 in host cells	^3^, ^13^, ^25^, ^26^, ^27^, ^28^, ^29^, ^30^, ^31^
		RNA‐dependent RNA polymerase (RdRp)	Enables the viral genome replication	Integral for preserving viral life	^32^, ^33^, ^34^, ^35^, ^36^, ^37^, ^38^, ^39^
		Human angiotensin‐converting enzyme (ACE2) receptor	Virus receptor	ACE2 binds to the RBD of SARS‐CoV‐2 spike and helps entry	^3^, ^25^, ^26^, ^40^, ^41^, ^42^, ^43^
		CD147 receptor	Virus receptor	Virus through CD147‐spike protein (SP) invades host cells	^27^, ^30^, ^44^, ^45^
		Main protease^46^ or 3‐chymotrypsin‐like protease (3CLpro)	Viral proteases, essential for viral infection cycle, establishment and replication	Cleaves the polyproteins (pp1a and pp1ab) of virus to produce the nonstructural proteins (nsp4‐16) responsible for the replication and transcription of the virus	^47^, ^48^, ^49^
		Papain‐like protease (PLpro)	Viral proteases, essential for viral replication	Cleaves at its LXGG recognition sites at nonstructural proteins nsp1, nsp2 and nsp3	^47^, ^50^, ^51^, ^52^, ^53^, ^54^, ^55^
		TMPRSS2	Plasma membrane‐associated host serine protease	Cleaves the viral spike protein to facilitate the fusion of viral and host membrane	^25^, ^56^, ^57^, ^58^, ^59^, ^60^
		Cathepsin L (CatL) Or CatB/L	Host cell proteases	S protein primes and facilitates the fusion of viral and endosomal membrane	^28^, ^57^, ^61^, ^62^, ^63^
		Furin‐like protease	Pat type I transmembrane protein and proprotein convertase genesis Relevance	Cleaves the S protein at the S1/S2 site in Golgi apparatus and the transmembrane serine protease 2 (TMPRSS2) at the S2′ site on cell surface	^28^, ^33^, ^56^, ^64^, ^65^, ^66^, ^67^, ^68^
		Tyrosine‐protein kinase receptor UFO (AXL)	Virus receptor	Specifically interacts with the N‐terminal domain of SARS‐CoV‐2 S with its extracellular Ig‐like domains and helps entry into pulmonary and bronchial epithelial cells	^13^, ^30^, ^44^, ^69^, ^70^
		NSP9 RNA binding protein (NSP9)	Leads to viral proliferation in host cells	Involves in the replication and translation which lead to viral proliferation in host cells	^50^
		Replicase Polyprotein 1a (RP1a)	The viral polyprotein large replicase polyprotein 1a, the nsps	Involves in the replication and translation which lead to viral proliferation in host cells e of action	^50^
		Envelope protein 2‐E	The smallest of the virus's four structural proteins	Involves in the replication and translation which lead to viral proliferation in host cells	^50^, ^71^, ^72^, ^73^
Potential therapeutic host targets essential for viral life or virus–host cell response	Host protein kinases and pathways	ABL	Involves in cell entry and replication	Plays critical role in viral egress	^74^, ^75^, ^76^, ^77^, ^78^, ^79^, ^80^, ^81^, ^82^
		SFKs	Play an important role in signal transduction, promote cell survival and motility	Play a critical role in the viral life cycle	^20^, ^83^, ^84^, ^85^, ^86^, ^87^
		AXL	The host receptors	Interacts SARS‐CoV‐2 with N‐terminal domain of Spike protein	^12^, ^13^, ^14^, ^20^, ^30^
		NAK	Regulates intracellular membrane trafficking	Regulates viral life cycle in the later stage	^88^, ^89^, ^90^, ^91^, ^92^
		EGFR	Activate effects cell growth, development, and survival	Plays a role in interstitial lung disease, drives development of fibrosis	^20^, ^93^, ^94^, ^95^, ^96^, ^97^, ^98^, ^99^, ^100^, ^101^
		PI3K/Akt/mTOR	Plays crucial roles in various cell biological processes, such as metabolism pathways, protein synthesis, cell proliferation, autophagy and cell growth, initiates the inflammatory response and producing cytokines	PI3K is over activated with virus entering, followed by phosphorylating AKT, which eventually causes activation of mTOR	^102^, ^103^, ^104^, ^105^, ^106^, ^107^, ^108^, ^109^, ^110^, ^111^
		CDKs	Are critical in cell cycle progression	SARS‐CoV‐2 inhibits CDK1/2 activity and gives rise to S/G2‐like phase arrest	^20^, ^112^, ^113^, ^114^, ^115^, ^116^, ^117^, ^118^, ^119^, ^120^, ^121^, ^122^
		Casein kinase 2	Matters in cell survival, neoplasia, and virus infection	Phosphorylates and modulates viral protein	^103^, ^112^, ^123^, ^124^, ^125^
		p38 MAPK	Essential for cell differentiation, apoptosis, and autophagy, links to the deregulation inflammatory responses	Induces the receptor‐mediated endocytosis for intrusive virus, boosts the synthesis of cytokines including IL‐6, TNF‐α, and β	^112^, ^126^, ^127^, ^128^
		JAK–STAT	A signaling pathway plays crucial roles in various biological process	Involves in cytokine storm triggered by SARS‐CoV‐2	^20^, ^129^, ^130^, ^131^, ^132^
		GSK‐3	A serine/threonine kinase	Be responsible for the phosphorylation of SARS‐CoV‐2 N‐protein on the serine residue in linker‐region	^121^, ^133^, ^134^, ^135^, ^136^, ^137^
	Immuno‐regulators	IL‐6	Proinflammatory cytokines	Leads to tissue damage and impairs NK cells antiviral activity	^138^, ^139^, ^140^, ^141^, ^142^, ^143^, ^144^
		IL‐1β	Proinflammatory cytokines	Causes CS and immune disruption	^145^, ^146^, ^147^, ^148^, ^149^
		IFN‐γ	Proinflammatory cytokines	Causes CS	^150^, ^151^
		TNFα	Proinflammatory cytokines	Excessive cytokine release and hyperinflammation	^152^, ^153^, ^154^, ^155^
		IL‐12/23	Proinflammatory cytokines	Excessive cytokine release and hyperinflammation	^156^, ^157^, ^158^, ^159^, ^160^, ^161^, ^162^
		IL‐17A	Proinflammatory cytokines	Causes CS and immune disruption	^163^, ^164^, ^165^, ^166^
		GM‐CSF	Be involved in autoimmune diseases and inflammatory diseases	Leads to and progression to ARDS	
		BRD2	A host protein controls transcription of interferon response in virus infection	Be involved in the CS response and the levels of ACE2 mRNA in COVID‐19	^167^, ^168^
		Thymosin alpha 1	A thymosin associated in the inflammation and cytokine storms	Destructs T‐cell immunity in patients with COVID19 and triggers cytokine storms	^169^, ^170^, ^171^, ^172^, ^173^, ^174^
		Immunoglobulin (Ig)	Regulates cytokine responses and immune cell functions	Prevents ARDS progression and improves the prognosis of COVID‑19	^175^, ^176^
		PI3K/Akt/mTOR	Involves in initiating the inflammatory response and producing cytokines	Triggers cytokine storms	^107^, ^177^, ^178^, ^179^, ^180^, ^181^
		NF‐κB	Links to the deregulation inflammatory responses	Boosts the synthesis of cytokines including IL‐6, TNF‐α,and β	^112^, ^128^, ^182^, ^183^, ^184^, ^185^, ^186^, ^187^
		JAK/STAT	The downstream of IL‐6 and other various cytokines	Involves in the CS	^130^, ^132^, ^188^, ^189^, ^190^
		GSK‐3	Incites systemic inflammation to worsen disease	Synthesizes the proinflammatory factors like IL‐6, IL‐1β, IL‐18, IFN‐γ, and TNF‐α	^136^, ^191^, ^192^, ^193^, ^194^, ^195^

### 2.1 Potential therapeutic viral targets associated with SARS‐CoV‐2

With a large RNA genome (30 kb), SARS‐CoV‐2 encodes many proteins (∼30) during the whole viral life cycle. There are the structural proteins, nonstructural proteins (nsps), and accessory proteins, which interact with numerous host cellular factors to conduct essential physiological roles.^32^ There are numerous potential molecular targets that are essential to virus’ four stages life cycle, including entry, replication, assembly, and release, and each has been investigated for development of repurposed or novel drug discovery. There are the structural viral Spike (S1) protein, which plays a critical role in the initial steps of pathogenesis, the viral entry receptors, such as the human angiotensin‐converting enzyme 2 (ACE‐2) receptor, tyrosine‐protein kinase receptor UFO (AXL) kinase, and host cell receptor CD147, which mediate viral infection by binding to the Spike (S) protein, the serine protease Transmembrane Serine Protease 2 (TMPRSS2), Furin and Cathepsin L (CatL), for Spike protein priming and facilitating the fusion of the viral and host membranes. Ongoing efforts to identify nonstructural proteins as antiviral targets for SARS‐CoV‐2 to date have focused mainly on several NSP proteins previously identified from SARS‐CoV and MERS‐CoV studies: NSP12 RdRp, which enables viral genome replication, NSP5 main protease,^46^ 3‐chymotrypsin‐like protease (3CLpro), and NSP3 papain‐like protease (PLpro).^50^ Several proteins, including Mpro, 3CLpro, and PLpro, are essential for the viral infection cycle and replication. In addition, the envelope protein can form cation channels, inducing host cell death in vitro and a cytokine storm in vivo^71^ (Figure 1). Moreover, some effective therapeutic, United States Food and Drug Administration (US FDA)‐approved agents with a well‐established safety profile have been identified to combat this viral disease with activity against key proteins. For COVID‐19 patients, there have been demonstrated, although variable, degrees of success with drugs including chloroquine (CQ)/hydroxychloroquine (HCQ), lopinavir/ritonavir, remdesivir, azvudine, favipiravir, molnupiravir, and azithromycin (Figure 1 and Table 1).

#### 2.1.1 Spike glycoprotein

Spike glycoprotein is the largest structural protein protruding from the surface of mature SARS‐CoV‐2 virions. It plays essential roles during the initial steps of pathogenesis, through mediating receptor recognition, cell attachment and fusion during viral infection, and these events ultimately lead to the virus entering the host cell. Some studies revealed the evolution between SARS‐CoV‐2 and SARS‐CoV with similarity in both structure and sequence; SARS‐CoV polyclonal antibodies are able to inhibit SARS‐CoV‐2 Spike‐mediated entry into cells.^3^, ^25^ This suggests potential for repurposing existing antibodies or drugs for urgent treatment of COVID‐19. Moreover, previously reported receptors of SARS‐CoV have also been confirmed to play a crucial role in SARS‐CoV‐2 entry, including ACE‐2, AXL, and the immunoglobulin superfamily member CD147 (Figure 1).^3^, ^13^, ^25^, ^26^, ^27^ To our knowledge, the Spike protein is cleaved into S1 and S2 subunits by proprotein convertases such as TMPRSS2, Furin, and CatL/CatB/L, during infectivity. The S1 subunit binds the host receptor and the S2 subunit anchors the S protein to the membrane and mediates membrane fusion with fusion peptide and other machinery necessary for a new infection.^28^

The surface location of the Spike glycoprotein renders it a direct target for host immune responses, making it the focus of many vaccine strategies as well as the main target of neutralizing antibodies. However, this protein is under strong selection pressure and is at high risk for undergoing mutagenesis, including mutations in the receptor binding domain (RBD) and mutations and deletions within the N‐terminal domain (NTD).^29^ Vaccines and neutralization antibodies specific for the Spike glycoprotein may be less effective for new subvariants evolving rapidly. This raises the risk for reinfection of these transmissible and immunity‐dodging subvariants, even in people who have already been infected or those who have recently been vaccinated. Several studies have reported that a single application of anti‐RBD antibodies may lead to a rise in generation of drug‐resistant mutations in the virus. Researchers isolated and identified a monoclonal antibody (4A8 mAb) in 10 COVID‐19 patients in remission. This monoclonal antibody was able to efficiently neutralize the COVID‐19 by targeting the NTD (and not the RBD motif, which could also generate a powerful neutralizing effect). This result suggests that mutations in the NTD are potential therapeutic targets for monoclonal antibody therapeutics against COVID‐19.^30^ In addition, if the two separate targeting antibodies against NTD and RBD, respectively, are combined, this approach could potentially override drug resistance to virus characterized by evolution of novel mutations; thus this is a potential direction for combination therapy.^31^ There remains an urgent need to develop novel therapeutic interventions targeting parts of the virus that are not as likely to evolve, such as the interaction between the Spike protein and its receptor rather than the Spike protein itself.

#### 2.1.2 Viral entry receptors: ACE‐2, AXL, and CD147

Spike proteins initiate cell entry by connecting viruses to cell membrane receptors, prior to catalyzing virus–host cell membrane fusions. ACE2 was the first receptor found for SARS‐CoV‐2 that was used to gain entry into the host cells. It has been previously shown that SARS‐CoV recognizes the entry receptor hACE2, and structural studies and biochemical experiments have also confirmed the binding of SARS‐CoV2 Spike protein to human ACE2 as the host receptor.^3^, ^25^, ^26^ The nanoluciferase‐based assay and in vitro binding measurements revealed that the Spike protein of the virus has a strong binding affinity to ACE‐2 receptors in the low nanomolar range, and the structural studies have shown that the Spike glycoprotein interacts with host cell epithelial ACE‐2 receptors with the RBD in the S1 subunit.^26^ Using structural analysis, they also identified the essential residues for ACE2 binding in the Spike RBD domain, suggesting a convergent evolution for improved binding to ACE2 from SARS‐CoV to SARS‐CoV‐2 RBDs.^26^, ^196^ Another study revealed that ACE2 expression was elevated in elderly individuals with impaired DNA repair capacity, suggesting age‐associated differences in SARS‐CoV‐2 infection and a novel approach for antiviral intervention.^40^ All findings suggest that ACE2 is a potentially useful target for developing selective and effective drugs against COVID‐19.

Considering the low expression of ACE2 in the human respiratory tract and high infectivity of COVID‐19,^41^, ^42^, ^43^ many Spike protein neutralizing human antibodies do not bind the RBD.^30^, ^44^ Two additional receptors were discovered: AXL and CD147 (Figure 1). Previous studies showed that pharmacological inhibition or genetic depletion of AXL is important for dengue viruses’ infection.^69^ Moreover, AXL is highly expressed in human pulmonary and bronchial systems and was identified in a complex with SARS‐CoV‐2 Spike protein via tandem affinity purification–mass spectrometry assay. Using both pseudotype and authentic virus of SARS‐CoV‐2, researchers confirmed that knocking out AXL or using soluble human recombinant AXL blocks SARS‐CoV‐2 infection in different cell models.^13^ Unlike the binding of ACE2 to the RBD, AXL likely binds the NTD of the Spike protein, which is consistent with effective neutralizing human antibodies that bind to the NTD rather than the RBD of S protein.^30^ These findings demonstrate an indispensable role for AXL in facilitating SARS‐CoV‐2 infection, suggesting combination treatments comprised of ACE2 and AXL inhibitors may be potentially efficacious therapeutic solutions for COVID‐19. Also supportive of a link between AXL and SARS‐CoV‐2 is an observed upregulation in AXL expression following SARS‐CoV‐2 infection in a non‐small cell lung cancer patient.^70^

CD147 is a transmembrane glycoprotein that has previously been reported to play a functional role in facilitating SARS‐CoV infection; it was confirmed to mediate SARS‐CoV‐2 entry by endocytosis and both loss and blocking of CD147 inhibited SARS‐CoV‐2 entry and amplification in different models.^27^, ^45^ Moreover, CD147 antibodies specifically inhibited infection and cytokine storm in different subvariants of COVID‐19, suggesting it could be a potential target for severe COVID‐19‐related pathogenesis.

#### 2.1.3 Proteases for Spike protein priming: TMPRSS2, Furin, and CatL/CatB/L

After the Spike glycoprotein initiates infection via binding to host receptors on cell surface, the latter membrane fusion requires proteolytic cleavages at two different sites (S1 and S2) by host cell proteases of S protein to develop a conformational flexibility; these activities reveal potential drug targets.^56^ There are several proteases identified, including TMPRSS2, Furin, and Cathepsin B and L (CatB/L), which play essential roles in S protein priming.^57^ TMPRSS2 is a type‐II transmembrane serine proteases, which cleaves the viral Spike protein at the S2 site to expose the fusion peptide for cell entry, and thus has an essential role in the virus lifecycle.^58^ SARS‐CoV employs the serine protease TMPRSS2 for S protein priming, and inhibition of serine proteases robustly block viral entry. Moreover, TMPRSS2 is also reported to be closely linked to the host cell receptor, ACE2, and forms a complex with it to enhance the SARS‐CoV virus entry; this correlates with proteolysis of S protein.^59^ A role of TMPRSS2 was recently identified in priming and mediating proteolytic maturation of the SARS‐CoV‐2 Spike protein for cell entry.^57^ An antisense‐mediated knockdown of TMPRSS2 led to robust inhibition of SARS‐CoV‐2 S protein activation in Calu‐3 human airway epithelial cells, demonstrating that TMPRSS2 is essential for S protein priming.^56^ The protease inhibitors, camostat mesylate and E‐64d, block SARS‐CoV‐2 infection of lung cells, and the effect is able to be rescued by directed expression of TMPRSS2.^25^ A Peptidomimetics that specifically inhibits TMPRSS2 activity demonstrated a potentially high level of prophylactic and therapeutic benefit against different subvariants (Alpha, Beta, Gamma, and Delta) of concern.^58^ Another study showed that in the airways of TMPRSS2‐knockout mice, Omicron infection efficiency is significantly reduced.^60^ This suggests TMPRSS2 is critically important for Omicron variant infection in murine airways. All of these findings support TMPRSS2 as an attractive pharmacological target for impeding entry of SARS‐CoV‐2 into host cells.

Furin‐like proteases are classified as a type I transmembrane protein and proprotein convertase that cleaves the precursors of a broad range of proteins, including cell surface receptors.^64^ Unlike SARS‐CoV and other coronaviruses, the Spike protein of SARS‐CoV‐2 is thought to be uniquely cleaved by this protease at a polybasic insertion. Recent studies have shown that Furin cleaves the S protein at the S1/S2 site in the Golgi apparatus and TMPRSS2 at the S2′ site on the cell surface.^28^, ^65^ Moreover, SARS‐CoV‐2 infection was strongly inhibited by the Furin inhibitor, MI‐1851, in human airway epithelial cells. A combination TMPRSS2 inhibitors with inhibitors of Furin exhibited more potent activity against SARS‐CoV‐2 than any monotherapy,^56^, ^66^ suggesting therapeutic potential of Furin for treatment of COVID‐19. In addition, Furin is considered to be an important mediator of T cells, including regulatory T cell (Treg) and T helper type 1 cells, through restricting TGFβ‐1 and interferon‐γ (IFN‐γ) signaling.^67^, ^68^ Therefore, inhibition of Furin may potentially suppress virus infection by twofold, via restraining viral entry and enhancing the immune response for viral clearance.^33^

The pH‐sensitive endosomal cysteine proteases CatB/L mediate the cleavage of an additional site internal to the S2 subunit of SARS‐CoV‐2 in the endosomal compartment, termed the “S2′ site,” which associates with ACE2. Membrane formation is then initiated by further release of the fusion peptide through ACE2‐mediated endocytosis.^28^, ^61^ CatB/L is involved in S protein activation and is present at the cell surface. Mediated S protein activation occurs at the plasma membrane, and CatB/L performs the cleavage and mediated activation occurs in the endolysosome.^28^ Recent studies have shown that the level of Cathepsin L was found to be elevated post‐SARS‐CoV‐2 infection in human cells and positively correlated with disease severity in ACE2 transgenic mice in vivo. Correspondingly, overexpression of Cathepsin L enhanced pseudovirus infection in human cells, and knockdown in vitro and application of Cathepsin L inhibitor drugs prevented infection both in vitro and in vivo.^62^ Previously, CatL‐selective inhibitors were reported to block coronavirus entry into host cells in vitro and in vivo for SARS‐CoV,^57^ and more recently were demonstrated to do so for SARS‐CoV‐2. Moreover, a combination of serine protease with CatL‐targeted inhibitors showed more potential therapy activity and safety than other available therapeutics in blocking coronavirus–host cell entry and replication, without disrupt the immune functions.^63^ These results suggest that Cathepsin L is a promising target for anti‐COVID‐19 drug development.

#### 2.1.4 RdRp

SARS‐CoV‐2 belongs to the class of positive‐sense RNA viruses. Its genome (30 kb) encodes two type proteins: (1) the structural, Spike (S), Nucleocapsid (N), Envelope (E), and Matrix (M) and (2) the nonstructural proteins (nsp1 up to nsp16), including RdRp (NSP12).^32^ RdRp is integral for preserving viral life, and it enables viral genome replication and transcription with cofactors nsp7 and nsp8.^32^, ^34^ RdRp enzymes are reported to be highly conserved in positive‐sense viruses,^35^, ^36^ and X‐ray crystallography and structural characterization of SARS‐CoV‐2 RdRp reveal that the active site of the RdRp is the most conserved and accessible region.^32^, ^35^ These results suggest that targeting this region for inhibition of viral replication may be an effective therapeutic approach, and also provide evidence for novel antiviral drug design and drug repurposing strategies.^37^, ^38^ In accordance with these findings, researchers found that pharmacological blockade of the activity of RdRp with compounds repurposed from US FDA‐approved databases robustly impaired the replication of different subvariants of SARS‐CoV‐2.^37^, ^197^ Further investigations are ongoing for preclinical and clinical studies to explore the potentials and effectiveness of these drugs and in the interest of developing safer and more potent therapeutics for treating COVID‐19.^39^

#### 2.1.5 The viral proteases: Mpro/3CLpro and PLpro

The replicase gene is a major component of the SARS‐CoV genome that encodes two large PPs (PP1a and PP1ab), which are cleaved into 16 NSPs. There are two types of cysteine proteases that act on these PPs to release the NSPs. The C‐terminal of these PPs (PP1a) is cleaved by Mpro or 3CLpro (corresponding to NSP5), and the N‐terminal of these PPs (PP1ab) is processed by PLpro (a domain within NSP3) into functional units.^50^ The nsps produced by Mpro/3CLpro includes NSP3, NSP9 and more importantly, the RdRp.^47^ These nsps are crucial for the replication and transcription of virus. In addition, compared with Spike protein, they are unlikely to develop drug‐resistant mutations rapidly and therefore are promising targets for antiviral drug development. Mpro is a critical drug target due to its importance in aiding the production of these proteins and establishment of the virus.^48^ With the aid of high‐resolution crystal structures, elucidation of active sites/pocket and substrate preferences, as well as high sequence similarity of both SARS‐CoV and SARS‐CoV‐2 3CLpro,^47^, ^49^ a panel of novel inhibitors developed against SARS‐CoV 3CLpro have been screened; this highlights the potential impact of crystallography‐guided fragment‐based drug discovery and computer‐aided virtual screening for drug repurposing.^47^, ^198^

Another potentially targetable SARS‐CoV‐2 protease is PLpro, which show high sequence homology (∼90%) to SARS‐CoV PLpro.^50^ PLpro recognizes the tetrapeptide LXGG motif between viral proteins nsp1/2, nsp2/3, and nsp3/4, thus leading to release of viral nsp1, nsp2, and nsp3 proteins; PLpro plays an essential role in cleavage and maturation of viral polyproteins, assembly of the replicase–transcriptase complex, and disruption of host responses.^51^ In addition, in vitro studies revealed the deubiquitinating activity of SARS‐CoV PLpro, efficiently disassembling mono‐polyubiquitin, di‐polyubiquitin, and branched‐polyubiquitin chains, and acting to remove ubiquitin (Ub) and Ub‐like (Ubl) protein IFN‐induced gene 15 (ISG15) from cellular proteins. SARS‐CoV‐2 PLpro was demonstrated to harbor deISGylating activity similar to SARS‐CoV PLpro,^52^, ^53^, ^54^ and contributes to cleavage of ISG15 from host proteins IFN responsive factor 3 (IRF3) and attenuates type I IFN responses to mediate evasion from host antiviral immune responses.^55^ With the high‐resolution structure of PLpro and active site Cys111 reported, as well as high sequence similarity with SARS‐CoV, designed and repurposed candidate drugs were found to inhibit the peptidase activity of PLpro and block SARS‐CoV‐2 replication, providing fundamental molecular and mechanistic insight into PLpro.^50^, ^54^ Moreover, the sequence, structure, and functional conservation of PLpro suggests that therapeutics targeting SARS‐CoV‐2 PLpro by drug repurposing of previously reported coronavirus inhibitors may be effective against COVID‐19.^54^, ^199^, ^200^

Other pathogenic targets involved in the SARS‐CoV‐2 life cycle include the envelope protein, the NSP9 RNA binding protein (NSP9), and the Replicase Polyprotein 1a (RP1a), which lead to viral proliferation in host cells.^50^ Envelope protein 2‐E is the smallest of the virus's four structural proteins. This class of protein has been found to have ion channel function in studies of other envelope proteins.^72^ In a recent report, researchers discovered that 2‐E can form cation channels that induce host cell death in vitro and a cytokine storm in vivo after infecting mice.^71^ Through activity screening and structure optimization, the newly developed 2‐E inhibitor is able to prevent and inhibit COVID‐19 infection in animal models. It was suggested that the envelope protein 2‐E could be considered as a potential COVID‐19 therapy target.^73^

### 2.2 Potential therapeutic host targets essential for viral life or virus–host cell response

Drugs that have been approved for COVID‐19 therapy are currently being developed based on antiviral targets that are necessary for different stages of the viral infection cycle.^32^, ^39^, ^56^, ^197^ However, direct viral targets continuously evolve due to changes in the genome, and thus novel therapies that were effective against early strains can lose their effectiveness against emerging variants. Repurposed drugs targeting proteins in the host cell that can interrupt virus–host cell interaction and that are necessary for viral propagation represent an alternative strategy that is potentially able to override antiviral drug resistance. Viruses, as cellular parasites, depend on host cell factors for completion of the viral life cycle; these factors are frequently shared by different viruses and thus mediate a broad range of effects. Since host factors are evolutionary conserved, targeting host cell proteins as an antiviral strategy would offer a higher genetic barrier to the emergence of viral resistance.^12^, ^14^, ^20^, ^88^, ^191^, ^201^

#### 2.2.1 Host protein kinases and pathways

All stages of the viral life cycle consume host resources, and cellular kinases in particular appear to be essential for the life cycle of the virus. Kinases are involved in transmission, pneumonia‐like symptoms, inflammation and fibrosis in coronavirus infection, and targeting them are now a significant effort of numerous pharmaceutical companies.^20^ Examples of potentially important antiviral kinase targets include Abelson tyrosine kinase (ABL), the SRC family of kinases (SFKs), AXL kinase, the Numb‐associated kinase (NAK), the tyrosine kinase receptor epidermal growth factor receptor (EGFR), PI3K/Akt/mTOR pathway signaling mediators, cyclin‐dependent kinases (CDKs), Casein kinase 2 (CK2), p38 MAPK, Janus kinase (JAK)–signal transducers and activators of transcription (STAT), and glycogen synthase kinase‐3 (GSK)‐3.^88^ ^,^ ^83^, ^84^, ^93^, ^94^, ^112^, ^129^, ^130^, ^133^ Many promising US FDA‐approved kinase inhibitors may be beneficial in treating the moderate and severe symptoms of COVID‐19 and can also be used to improve the efficacy of other antiviral drugs or to tailor therapy against SARS‐CoV‐2. Some are now being tested in clinical studies to see if they can be a viable treatment to overcome respiratory distress or complications in COVID‐19 patients.^14^, ^74^, ^131^, ^177^, ^178^, ^182^, ^188^, ^202^ Our colleagues previously have predicted and discussed many as potential targets associated with the coronavirus virus infection and symptoms of COVID‐19 and prioritized a panel of repurposed kinase inhibitors as candidates for therapy.^10^, ^14^ Here, we provide a brief introduction to this effort as well as a summary of progress made in this area.

##### ABL

ABL is a nonreceptor tyrosine kinase that is involved in cell proliferation, survival, migration and stress responses.^75^, ^76^ This kinase is conserved in variety of viruses, such as Ebola virus, Coxsackie virus, and vaccinia virus, and it has been implicated in several stages of the viral life cycle.^77^, ^78^, ^79^ Studies have demonstrated that ABL kinase is involved in cell entry and replication of the SARS‐CoV‐1 virus and was recently identified as a therapeutic target for combating SARS‐CoV‐2 infection. Moreover, a case reported that with dasatinib (BMS‐354825) (Src/ABL tyrosine kinase inhibitor) treatment, a chronic myeloid leukemia (CML) patient went into remission after experiencing severe SARS‐CoV‐2 infection and symptoms; this suggests that ABL kinase may play a critical role in viral egress and is a potentially attractive target for coronavirus replication.^80^ Researchers identified inhibitors by screening approved or clinical drugs as potential antiviral drugs previously used for SARS‐CoV‐1 and MERS‐CoV, and identified imatinib, dasatinib and nilotinib.^74^, ^81^, ^82^ Pharmacological inhibition of ABL kinase activity through those candidates robustly inhibited the SARS‐CoV viral load, thus supporting the notion that ABL kinase is a potential therapeutic target for COVID‐19.

##### SFKs

The SFKs is a class of nonreceptor tyrosine kinases that play an important role in signal transduction, and regulate a number of biological processes that promote cell survival and motility, as well as inflammatory responses.^20^ This family includes nine members: Src, Yes, Fyn, Fgr, Lck, Hck, Blk, Lyn and Yrk.^20^ These kinases are activated independent of ABL kinases.^85^ SFKs have recently emerged as critical mediators between the infrastructure of host cells and viral demands. SFKs have been shown to play a critical role in the viral life cycle. For example, studies revealed that MERS‐CoV load was significantly reduced after siRNA knockdown of Lyn and Fyn, which suggests the importance of these protein kinases for MERS‐CoV replication.^83^ Yes was shown to reduce West Nile virus load by acting on viral replication cycle, assembly and release.^84^ Moreover, pharmacological inhibition of the SFK signaling pathway led to inhibition of MERS‐CoV, HCV77, and dengue virus infection and replication in vitro,^84^, ^86^, ^87^ and some family members of SRC kinases have been linked to SARS‐CoV replication.^20^ These findings suggest that these kinases may be attractive targets for COVID‐19 therapy.

##### AXL

AXL kinase is an identified host receptor of SARS‐CoV‐2 that interacts with the NTD of Spike protein.^13^, ^30^ Previously, our group stratified a panel of kinase inhibitors according to their predicted ability to exhibit anti‐SARS‐CoV‐2 activity, including the importance of AXL‐related kinases in virus entry into cells.^14^ They selected and investigated kinase inhibitors based on two databases: (1) KINOMEscan from the Harvard Medical School Library and (2) ChEMBL, which identified potential kinase targets in SARS‐CoV, MERS‐CoV, and other related viruses. Among those identified were several tyrosine kinase inhibitors targeting AXL and AXL‐related kinases. Genetic deletion of AXL, a target of gilteritinib and nintedanib, led to robust anti‐infectivity of different subvariants of SARS‐CoV‐2, including the Omicron in both Vero (African green monkey) cells and human lung adenocarcinoma (A549‐ACE2) cells. AXL has been implicated in pneumonia, cytokine storm, systemic inflammation, and lung fibrosis, and thus repurposing of AXL inhibitors would be anticipated to possibly exhibit a dual, clinically beneficial effect through preventing viral entry and reducing the related inflammatory response and cytokine storm.^12^, ^14^, ^20^ These results support a role for AXL in SARS‐CoV‐2 infection and warrant further research into drug development of AXL as a therapeutic for COVID‐19.^12^

##### NAK

The NAK family consists of four members: (1) the adaptor‐associated kinase 1 (AAK1), (2) cyclin G‐associated kinase, (3) BMP‐2 inducible kinase (BIKE/BMP2K), and (4) serine/threonine kinase 16 (STK16), which act to regulate intracellular membrane trafficking.^89^, ^90^ AAK1 and GAK have been linked to the viral entry through mediating and promoting endocytosis.^91^, ^92^ Recently, using strategies integrating virology, genetic and pharmacological approaches, Karim et al.^88^ discovered that all four NAKs, including BIKE and STK16, are required in SARS‐CoV‐2 infection. Moreover, their time‐of‐addition experiments further clarified that NAKs participate in regulating viral life cycle in the later stage, such as assembly or egress, but not RNA replication.^88^ Moreover, combination with inhibitors of AAK1/BIKE and GAK led to a synergistic effect against SARS‐CoV‐2 in vitro.^88^ These crucial roles of NAKs in SARS‐CoV‐2 make them a potential host cell kinase target for SARS‐CoV‐2 treatment.

##### EGFR

EGFR and its activation affect cell growth, development, and survival.^95^, ^96^ Studies have shown that it plays a role in interstitial lung disease and is believed to drive development of fibrosis through interaction between EGFR and TGF‐beta signaling.^97^, ^98^ It is also associated with the infection of various viruses.^99^, ^100^ Inhibiting EGFR signaling may prevent an excessive lung fibrotic response to SARS‐CoV and other respiratory virus infections.^94^ Though the role of EGFR signaling in the development of lung fibrosis is controversial, pharmacologic inhibition of EGFR has led to robust reduction of TGF‐beta1 induction of fibrosis both in vitro and in vivo in a variety of animal models.^101^, ^197^ Moreover, studies have revealed overexpression of EGFR in the lung tissue from COVID‐19 patients.^93^ Despite SARS‐CoV‐2 being cleared, EGFR signaling remained actived.^94^ A deficit of STAT1 could be induced by SARS‐CoV‐2, thus leading to EGFR overexpression in epithelial cells followed by activation of STAT3.^20^ Therefore, targeting EGFR may be effective against SARS‐CoV‐2 via both relieving pulmonary symptoms as well as suppressing fibrosis. This suggests that EGFR could be an attractive target for COVID‐19 treatment.

##### PI3K/Akt/mTOR

SARS‐CoV‐2 relies on regulating host metabolism pathways to meet its need to produce as many virus particles as possible. Among those host signaling pathways, the phosphatidylinositol 3‐kinase/protein kinase B/mammalian target of rapamycin (PI3K/Akt/mTOR) cell signaling pathway has drawn attention. The PI3K/Akt/mTOR pathway regulates and incorporates various signaling processes in cells, including cell proliferation. Recently, studies have shown that activation of the PI3K/Akt/mTOR signaling pathway is involved in the proliferation and replication of a variety of viruses, including hepatitis C virus (HCV), West Nile virus, and influenza A virus.^102^, ^103^, ^104^ Pathway overrepresentation and functional network analysis revealed that several members of the signaling pathway, including AKT, mTOR, and the Ras/Raf/MEK/ERK, are upregulated during infection with a variety of different viruses, including MERS and SARS‐CoV‐2.^105^, ^106^ When virus enters into the host cell, PI3K is overactivated followed by phosphorylation of AKT, which eventually causes activation of mTOR.^107^ One study reported that Akt inhibition downregulates the expression of the host receptor ACE2.^108^ mTOR, a serine‐threonine protein kinase, plays crucial roles in various cell biological processes, such as metabolism, protein synthesis, cell proliferation, autophagy, and cell growth.^109^ Moreover, mTOR is also involved in initiating the inflammatory response and producing cytokines.^107^ Pharmacological inhibition of mTOR or RAF led to significant inhibition of MERS‐CoV or influenza A infection.^105^, ^110^ Recent studies also revealed that RAS signaling inhibition can impair the replication of SARS‐CoV‐2.^111^ These results suggest that members of this signaling pathway may be exploited as potential therapeutic targets for SARS‐CoV‐2 treatment.

##### CDKs

CDKs are a family of serine/threonine kinases that include 20 members.^113^ Linked to both extra and intracellular signals, CDKs are critical for cell cycle progression, and thus blocking of CDK activity could be of benefit to cancer patients.^114^, ^115^, ^116^ In addition, CDKs have also been targets of various infectious diseases because their expression and function are affected by infection with multiple viruses, such as human immunodeficiency virus (HIV), herpes simplex virus (HSV), Zika virus, and hepatitis B virus (HBV).^117^, ^118^, ^119^, ^120^ It has been demonstrated that the highly conserved N protein plays an important role in regulating CDKs.^121^ In SARS‐CoV‐1, cyclin‐CDK4, and cyclin A/E‐CDK2 complexes are restrained by N protein. SARS‐CoV‐2 has been shown to inhibit CDK1/2 activity, giving rise to S/G2‐like phase arrest.^112^ Given the essential roles CDKs play in viral infection, they may be considered as potential targets for SARS‐CoV‐2 therapy. Moreover, studies have also shown that repurposed approved or clinical CDKs inhibitors, such as CK2 inhibitors, could be new strategy to combat SARS‐CoV‐2 infection and transmission in vitro and some of these candidates are now in clinical trials.^20^, ^122^

##### CK2

CK2 is a highly pleiotropic and constitutively active Ser/Thr kinase that is important for cell survival, neoplasia and virus infection.^123^ CK2 participates in several infectious diseases by phosphorylating and modulating viral proteins, including those of HCV, vesicular stomatitis virus, HIV,^103^ human papilloma virus, and HSV‐1.^124^ One study revealed that CK2 may be involved in downregulating the ability of host cells to produce IFN during viral infection; as such, blocking CK2 may be considered an important antiviral therapeutic strategy.^125^ Studies have shown that in cases of SARS‐CoV‐2 infection, CK2 was activated and directly targeted by the N protein and facilitated the rapid spread and movement of the virus between cells.^112^ Given the essential roles of CK2 in viral infection, CK2 is a putative therapeutic target for SARS‐CoV‐2.

##### p38 MAPK

The p38 MAPK signaling pathway can be triggered by diverse factors, including environmental stress, viral infection and cytokine storm. This pathway is essential for cell differentiation, apoptosis and autophagy. In SARS‐CoV‐1, p38 is directly involved in viral proliferation by inducing the receptor‐mediated endocytosis for intrusive virus.^126^ The hypothesis that p38 signaling pathway can also be similarly activated by SARS‐CoV‐2 was verified by a recent study.^127^ Death of COVID‐19 patients is often associated with an overwhelming inflammatory response and ARDS. Deregulation of inflammatory responses linked to p38 MAPK was originally discovered in association with SARS‐CoV‐1, which leads to several serious diseases.^112^ Moreover, the synthesis of cytokines, including interleukin (IL)‐6, tumor necrosis factor (TNF)‐α and β, is boosted after the activation of p38 MAPK signaling pathway.^128^ Taken together, p38 MAPK is considered as a potential target for effective treatment of COVID‐19.

##### JAK–STAT

SARS‐CoV‐2‐infected patients present some common symptoms, including fever, dry cough or dyspnea. However, the severe and life‐threatening pathology, such as failure of organs and tissue injuries, are majorly due to cytokine storm resulting from a virus‐driven hyper‐inflammatory response. JAK–STAT are among a large number of pathways involved in cytokine storm triggered by SARS‐CoV‐2.^132^ The JAK family is composed of JAK1, JAK2, JAK3, and tyrosine kinase 2 (Tyk2) proteins. The STAT family consists of STAT1, STAT2, STAT3, STAT4, STAT5a, STAT5b, and STAT6. The JAK–STAT signaling pathway plays crucial roles in various biological process, including apoptosis, cell cycle progression, differentiation, and hyperactivated immune response.^20^ As viruses invade host cells, the latter initiate various downstream regulatory factors to generate IFN for vigilance mechanisms. When these cytokines bind to receptors, the activated receptors phosphorylate JAK and then STAT proteins that subsequently translocate into the nucleus to express inflammatory factors.^131^ Moreover, the JAK2/STAT3 pathway plays critical roles in systemic inflammation during coronavirus infection.^129^, ^130^ Overall, alleviating the potentially fatal symptoms stemming from excessive inflammatory response by targeting JAK–STAT is an attractive therapeutic approach to treatment of COVID‐19.

##### GSK‐3

GSK‐3 is a highly conserved serine/threonine kinase that plays a role in metabolism, inflammatory response, and cell proliferation.^134^ GSK‐3 is important for the viral life cycle by being associated with the N‐protein of SARS‐CoV‐1.^133^ N protein, highly conserved among the species revealed by protein sequencing, acts to support and protect the viral genome, replication, and ensure proper transmission.^121^ GSK‐3 has been shown to be a crucial kinase responsible for the phosphorylation of N‐protein on the serine residue in linker‐region.^133^ The activated GSK‐3 protein in the viral infected cell is able to degrade the nuclear factor erythroid 2‐related factor, which contributes to excessive oxidative stress.^135^ Beyond regulation of viral replication and initiating oxidative stress, GSK‐3 also incites systemic inflammation to worsen disease through synthesizing proinflammatory factors, such as IL‐6, IL‐1β, IL‐18, IFN‐γ, and TNF‐α.^136^ These findings suggest that targeting GSK‐3 may provide therapeutic benefit to COVID‐19 patients.

##### BRD2

Another potential host therapeutic target is BRD2, a member of the BET (bromodomains and extra terminal domain) family, which can regulate gene transcription through binding acetylated histones.^203^ BRD2 is closely linked to infection by various types of viruses, including Kaposi's sarcoma associated‐herpesvirus, murine herpesvirus68, Epstein–Barr virus, and murine leukemia virus.^204^, ^205^, ^206^, ^207^, ^208^, ^209^ Since the infection of host cells by COVID‐19 mainly depends on the RBD domain of the Spike protein, researchers used RBD binding as a phenotypic readout in a high‐throughput CRISPRi screen to systematically discover significant factors regulating viral infection in host cells.^167^ The results demonstrated that knocking out the BRD2 gene effectively prevented RBD binding. Another group used a proteomics study to determine and validate the associative relationship between SARS‐CoV‐2 and BRD2.^210^ Cell‐based infection assays and in vivo models confirmed the antiviral effects of inhibiting BRD2.^167^ Knocking out BRD2 or inhibiting BRD2 with small molecules strongly inhibited the infection and growth of the virus, and also resulted in marked downregulation of genes involved in the type I IFN response. These findings suggest that BRD2 is a potent inhibitory target of COVID‐19 infection. Moreover, BRD2 is essential for ACE2 transcription in human lung epithelial cells, and BRD2 inhibitors are currently being evaluated in clinical trials for SARS‐CoV‐2 infection therapy.^167^

Additional potential kinases exist that could be explored as targets for COVID‐19 remission. For example, molecular targeting of the ataxia telangiectasia and Rad3‐related protein, which is associated with DNA‐damage response signaling pathways, has been shown to inhibit SARS‐CoV‐1, SARS‐CoV‐2, and MERS‐CoV infection.^137^ Collectively, these key promising kinase targets could constrain coronavirus infection and symptoms and lead to effective drug repurposing for the treatment of COVID‐19.

#### 2.2.2 Immunoregulators associated with virus–host response

Other potential targets associated with virus–host response have been identified in clinical studies. The complex immunopathological manifestations of COVID‐19 include lymphopenia or exhaustion (markers are PD‐1, Tim‐3, or NKG2A), dysregulation of monocytes and macrophages (phenotypic shift and increased numbers), neutropenia (increased precursors in peripheral blood), antibody‐dependent enhancement (which may enhance entry of SARS‐CoV‐2), reduced or delayed IFN‐I response (impedes viral clearance and induces excess inflammation) and more alarmingly, cytokine storm, characterized by increased levels of proinflammatory mediators that correlate with worse prognosis in patients with severe infection.^22^, ^46^, ^211^, ^212^, ^213^, ^214^ These manifestations are associated with many signaling pathways, including JAK/STAT signaling, NF‐κB and MAPK pathways, which induce the expression of proinflammatory cytokines and paradoxical hyperinflammation in COVID‐19. The key modulators related to these pathways could potentially be new therapeutic targets.

Extensive evidence from preclinical and clinical studies suggests that cytokine storm syndrome may be an important mechanism responsible for this respiratory disorder.^24^ An imbalance in host immune regulation leads to an overwhelming release of inflammatory mediators, such as TNF‐α, IFN‐α, IFN‐γ, GM‐CSF, IL‐1β, IL‐2, IL‐6, IL‐17A, and monocyte chemotactic protein‐1 (MCP‐1), which cause potentially life‐threatening cytokine storm associated with extensive lung injury in SARS‐CoV‐2‐infected patients.^23^ For example, as a major cytokine effector of the host immune response to viral infection, IFN‐γ serves as an immunomodulator by promoting macrophage‐mediated antigen phagocytosis and mediating the clearance of infected cells by NK cells, thus limiting viral transmission.^215^ IFNs are also often used to treat viral diseases such as hepatitis B and C.^216^ IFNs are implicated in restraining SARS‐CoV‐2 infection; however, they may also contribute to severe symptoms; thus, blocking IFNs maybe beneficial for treatment.^217^ Recent studies identified other targets involved in cytokine storm, including AXL, ABL, p38 MAPK, PI3K/Akt/mTOR, JAK2/STAT3, and GSK‐3. These proteins were shown to play critical roles in systemic inflammation, fibrosis formation, and pneumonia, thus making them attractive targets for the fight against infection.^12^, ^107^, ^128^, ^129^, ^136^, ^202^ Thus, repurposing of kinase inhibitors offers great potential for development of potent drugs for treatment of COVID‐19 disease.

Therapy approaches targeting virus–host responses, including immunomodulatory therapies (including the use of monoclonal antibodies, IFNs, thymosin, immunoglobulins) may be helpful in alleviating SARS‐CoV‐2 symptoms; other options, such as stem cell‐based therapy and blood purification therapy, may also provide clinical benefit.^218^, ^219^, ^220^

## 3 REPURPOSING CLINICALLY AVAILABLE DRUGS AND THERAPIES FOR PATHOGENIC TARGETS

**FIGURE 2:**
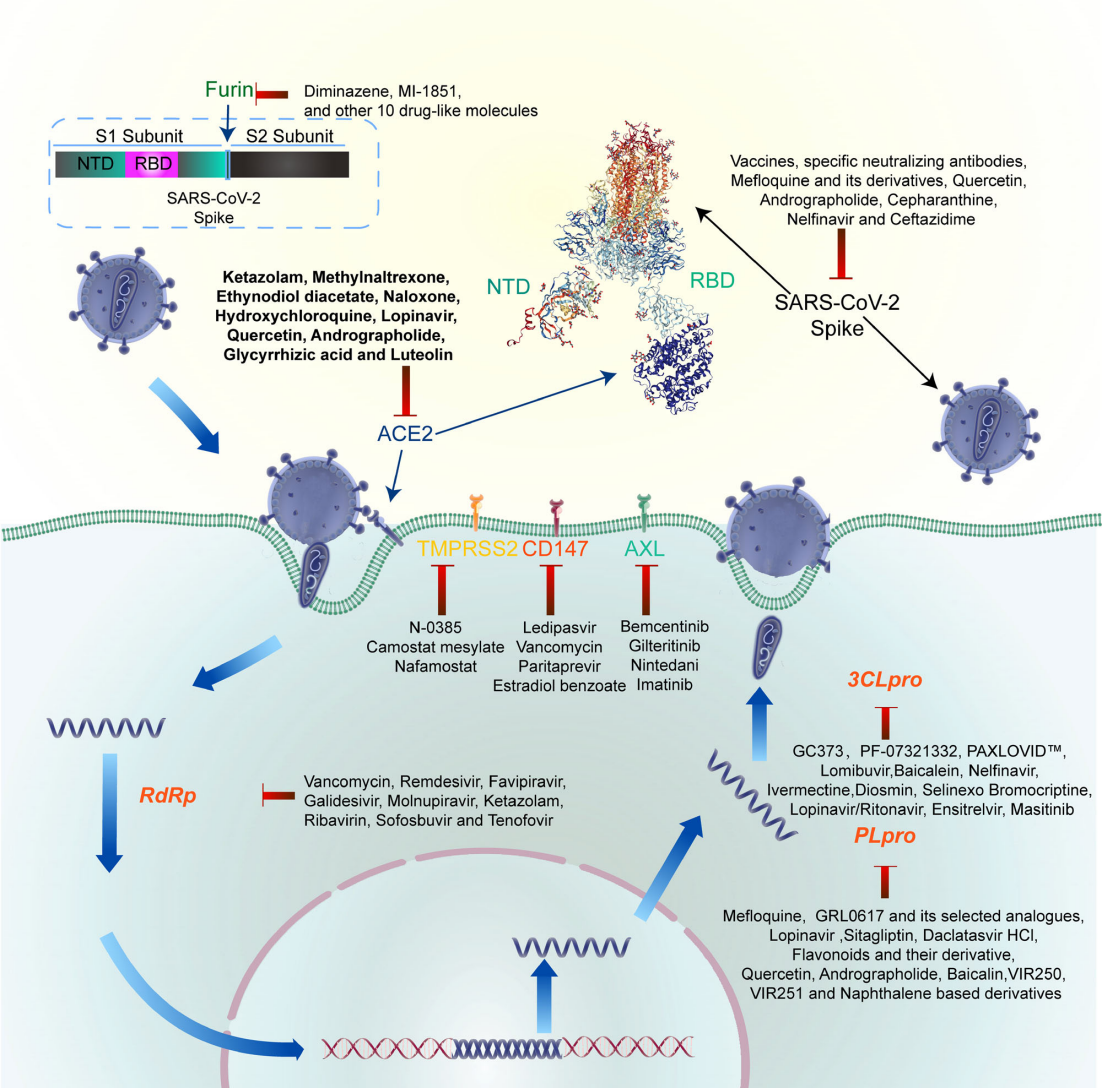
Repurposed approved/clinical drugs for potential pathogenic targets associated with SARS‐CoV‐2 viral life cycle. SARS‐CoV‐2 uses the spike protein to recognize human cells and induce the fusion of the viral and human host cell membranes through binding to receptors. These high‐affinity interactions are essential for viral entry and are therefore important targets in the treatment of COVID‐19. After priming by TMPRSS2, the virus enters the cytoplasm through CD147‐ or ACE2‐mediated endocytosis, releases the viral genetic material, and then replicates (via RdRp, 3CLpro, and PLpro). This is accompanied by protein synthesis, viral particle assembly and release and infection of adjacent cells. Repurposed approved/clinical drugs interfere with different viral targets and demonstrate effectiveness against coronaviruses. These are shown as indicated.

**FIGURE 3:**
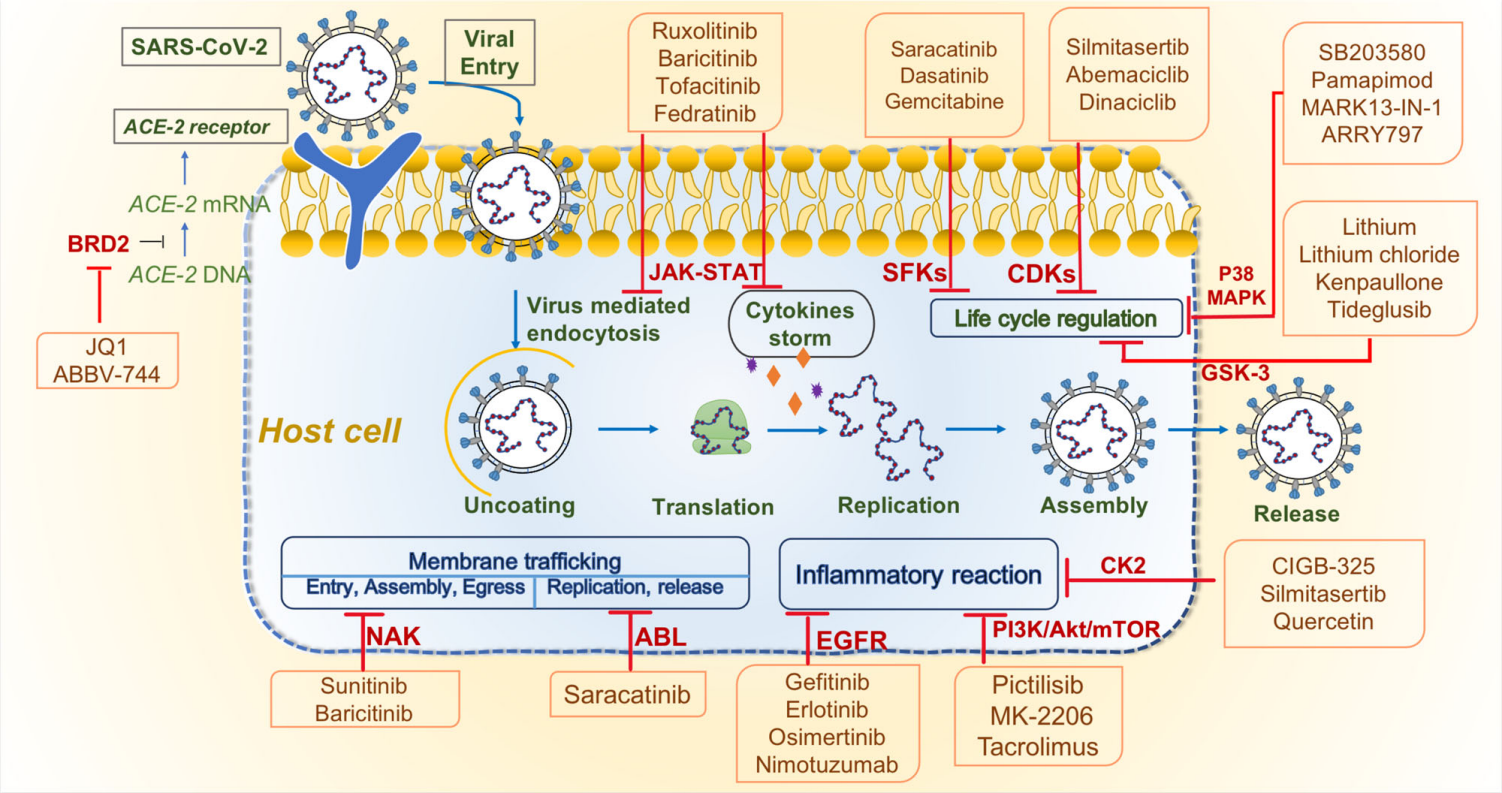
Repurposed approved/clinical drugs for potential pathogenic targets associated with host protein kinases and pathways essential for viral life or virus–host cell response. Host cell protein kinases and pathways play crucial roles in various stages of the virus's life cycle, and repurposed drugs may be developed into antiviral agents. Ruxolitinib and baricitinib, inhibitors of the JAK–STAT pathway, potentially inhibit both viral‐mediated endocytosis and cytokine storm. Approved drugs targeting NAK or ABL, including sunitinib, baricitinib and saracatinib, are potential therapies for COVID‐19 through prevention of membrane trafficking. Repurposed inhibitors of SFKs, CDKs, P38 MAPK and GSK‐3, such as saracatinib, silmitasertib, SB203580 and lithium, are under investigation for COVID‐19 based on their ability to regulate the viral life cycle. Gefitinib, pictilisib and CIGB‐325, inhibitors of EGFR, PI3K/Akt/mTOR, and CK2, may provide clinical benefit through reduction of the inflammatory response. JQ1 and ABBV‐744, two inhibitors of BRD2 that block viral entry by inhibiting ACE‐2 transcription, are currently being evaluated in clinical trials for SARS‐CoV‐2 infection.

**FIGURE 4:**
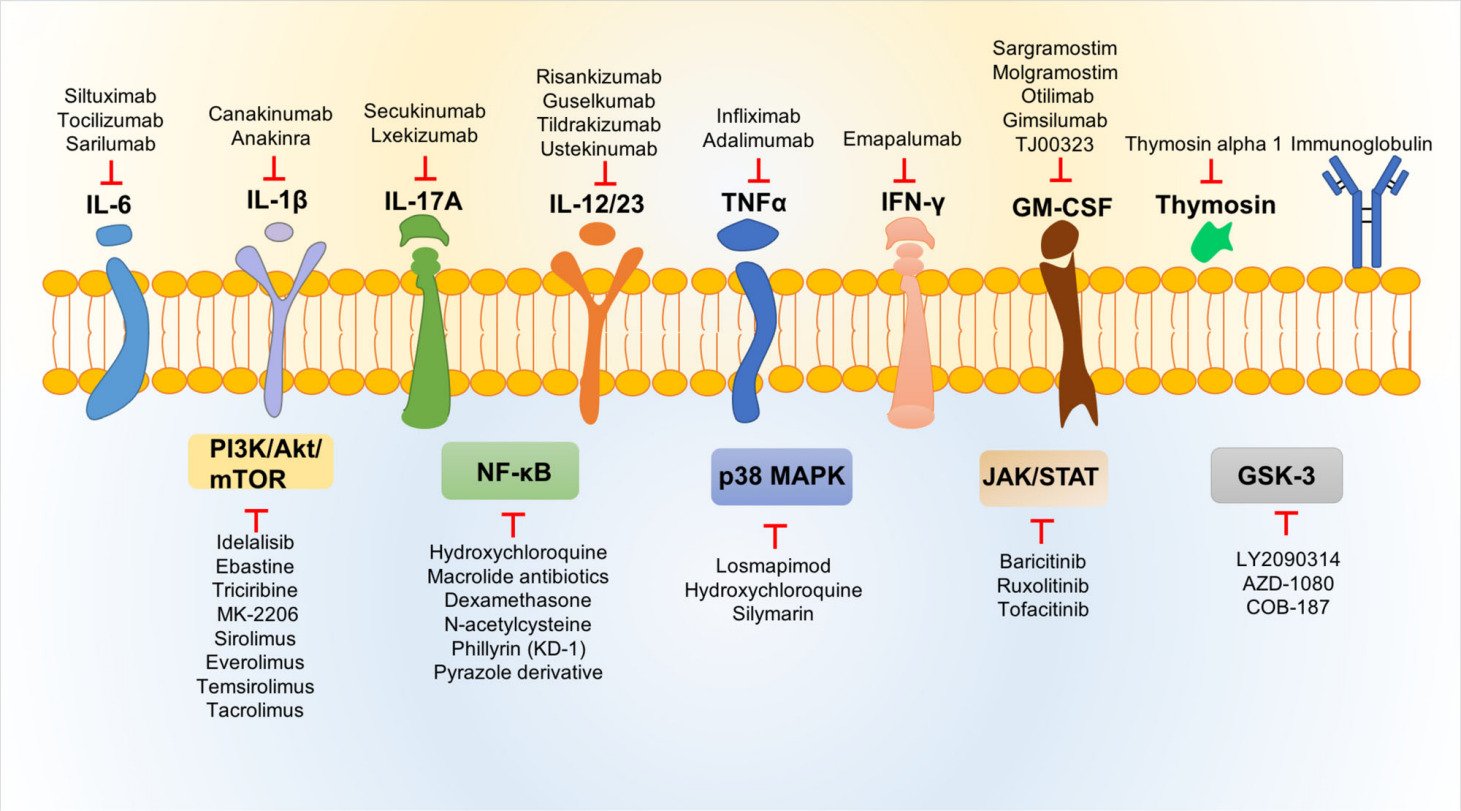
Repurposed approved/clinical drugs for potential pathogenic targets associated with the immunoregulators associated with virus–host response. Repurposed approved/clinical drugs have been investigated as treatments for COVID‐19 based on their ability to target inflammatory cytokines, immune‐based factors, and related signal pathways. Drugs targeting immunoregulators, such as anakinra and tocilizumab, have shown high efficacy in preclinical and clinical studies for SARS‐CoV‐2. Anakinra is an IL‐1 receptor antagonist, and tocilizumab is an IL6R antibody. There are currently over 30 clinical trials underway for evaluating the efficacy of these compounds in mono‐ or combination treatments, according to the Clinical Trials.gov. Anti‐inflammatory treatments, including intravenous immunoglobulin, thymosin, may have potential as treatments for COVID‐19.

Drug discovery is a lengthy, complex, and costly process, from conception to availability in the market, with a high degree of uncertainty. Among different approaches that have been proven effective for combating COVID‐19, a drug repurposing strategy is a more economical and faster approach than the traditional drug discovery and development process. The concept of identifying potent molecules from drug libraries that have been approved or are in clinical trials, or compounds that have been tested against conserved targets in coronaviruses previously, has become accelerated, effective, and feasible ways to identify drugs that can prevent COVID‐19.^11^, ^12^, ^14^, ^15^ Currently, combined with experimental and computational drug repurposing strategies, our scientific community is largely focused on identification of potential new drugs that can be utilized as potential therapies for SARS‐CoV‐2 infection^11^, ^12^, ^14^, ^15^ (Table 2 and Figures 2, 3, 4).

**TABLE 2:** Summary of repurposed approved/clinical drugs for pathogenic targets that have been vetted for safety and that are more accessible for COVID‐19 treatment, including their drug mechanism‐of‐action.

Pathogenic targets	Repurposing strategies	Repurposed drugs	Mechanisms of action	References
Spike glycoprotein	Drug repurposing strategy with screening of approved antiviral/antiparasitic/antiprotozoal drugs	^1^ **Specific neutralizing antibodies, quercetin, andrographolide, cepharanthine**, ^2^ *Mefloquine and its derivatives, nelfinavir, ceftazid ime*	Target the viral spike protein, prevent Sp‐ACE2 binding or act as an entry inhibitor in a prophylactic role	^25^, ^221^, ^222^, ^223^, ^224^
RNA‐dependent RNA polymerase (RdRp)	In silico drug repurposing strategy screening compatible inhibitors and a molecular docking study of US FDA‐approved drugs	**Remdesivir, favipiravir, galidesivir, molnupiravir (MK‐4482, EIDD‐2801), ketazolam, ribavirin, sofosbuvir, tenofovir,** *vancomycin*	Inhibit viral replication	^225^, ^226^, ^227^, ^228^, ^229^, ^230^, ^231^, ^232^
Human angiotensin‐converting enzyme (ACE2) receptor	A high‐throughput virtual screening and molecular docking	**Ketazolam, hydroxychloroquine, lopinavir, quercetin, andrographolide, luteolin,** *glycyrrhizic acid*	Interfere the binding of S protein to ACE2 or prevent the entering of the virus into the host cells	^223^, ^226^, ^227^
CD147 receptor	US FDA‐approved drugs repurposing strategy with virtual screening and molecular dynamic simulations	*Ledipasvir, vancomycin, estradiol benzoate*	Prevent the entering of the virus into the host cells	^226^
Main protease^46^ or 3‐chymotrypsin‐like protease (3CLpro)	An in‐cell protease assay (ICP) that measures the protease activities based on the subcellular localization of a cleaved fluorescent protein in live cells, fluorescence resonance energy transfer (FRET)‐based SARS‐CoV‐2 M pro enzymatic assay to explore a library of known protease inhibitors, structure‐based virtual and high‐throughput screening	**PF‐07321332, PAXLOVID™, luteolin, chloroquine, ebselen, nelfinavir, ivermectine (NCT04668469), diosmin (NCT04452799), selinexor (NCT04349098), lopinavir/ritonavir, flavonoids and their derivatives, andrographolide, luteolin, ensitrelvir, masitinib, quercetin,** *boceprevir, carmofur, GC373, MG‐101, lycorine HCl, nelfinavir mesylate, lomibuvir, BMS‐707035, baicalein, GC‐376, calpain inhibitors II and XII, elbasvir, baicalin*	Covalently or noncovalently modifying the catalytic Cys145 of M pro, blocking the substrate binding at the active site, inhibit the protease activity, blocking virus polyprotein processing and viral replication cycle	^224^, ^233^, ^234^, ^235^, ^236^, ^237^, ^238^, ^239^, ^240^, ^241^, ^242^, ^243^, ^244^, ^245^, ^246^, ^247^
Papain‐like protease (PLpro)	An in‐cell protease assay (ICP), HyCoSuL (Hybrid Combinatorial Substrate Library), virtual screening of the US FDA‐approved drug library, docking, and molecular dynamics simulation studies	**Mefloquine, lopinavir, sitagliptin, flavonoids and their derivative, quercetin, andrographolide,** *GRL0617 and its selected analogs, daclatasvir HCl, baicalin, VIR250, VIR251, naphthalene‐based derivatives*	Inhibit virus replication by shutting down the active site of PLpro, might decrease the activity of PLpro by binding at an allosteric site	^239^, ^248^, ^249^, ^250^, ^251^, ^252^, ^253^, ^254^
TMPRSS2	A virtual screening of the US FDA‐approved drug library	**Camostat mesylate, nafamostat**, N‐0385 ^3^ *, aerosolized aprotinin*	Act as TMPRSS2 inhibitors, impair the proteolytic activation of virus spike protein, block viral–cell membrane fusion and entry	^25^, ^58^, ^255^, ^256^
Cathepsin L (CatL) Or CatB/L	A high‐throughput screening of US FDA‐approved drugs	*E‐64d*	Impairs S protein priming/proteolysis in endosome membrane and viral entry	^25^, ^255^
Furin‐like protease	Structure‐based virtual screening, post‐screening biochemical assay, a chemoinformatic approach of compounds from the ChEMBL database	*Diminazene*	Cleave the SARS‐CoV‐2 S protein	^56^, ^257^, ^258^
Tyrosine‐protein kinase receptor UFO (AXL)	Combining the KINOMEscan‐LINCS biochemical kinase profiling database with the ChEMBL database of targets associated with SARS‐CoV	**Bemcentinib, gilteritinib, nintedanib, imatinib**	Specifically inhibit AXL activity and blocks entry into pulmonary and bronchial epithelial cells	^12^, ^70^, ^259^, ^260^
ABL	Screening from US FDA‐approved drugs	*Saracatinib*	Restrain the life cycle and viral proliferation of SARS‐CoV‐2	^83^
SFKs	Screening from US FDA‐approved drugs	**Dasatinib**, *saracatinib, gemcitabine*	Restrain the life cycle and viral proliferation of SARS‐CoV‐2	^83^, ^261^, ^262^
NAK	Screening from US FDA‐approved drugs	**Baricitinib,** *sunitinib*	Block viral entry, assembly, and traffic	^69^, ^263^, ^264^, ^265^, ^266^
EGFR	Screening from 24 US FDA‐approved drugs	**Nimotuzumab,** *gefitinib, erlotinib, osimertinib*	Reduce inflammation and fibrosis in severe and moderate COVID‐19 patients	^93^, ^122^, ^267^, ^268^, ^269^
PI3K/Akt/mTOR	Screening from US FDA‐approved drugs	**Rapamycin, metformin, tacrolimus,** *pictilisib, VPS34‐IN1, MK‐2206, omipalisib, SF2523, sorafenib, ionafarnib*	A combined mechanism entailing downregulation of excessive inflammatory reactions, cell protection, and antiviral effects	^105^, ^106^, ^179^, ^270^, ^271^, ^272^, ^273^, ^274^, ^275^, ^276^
CDKs	Quantitative mass spectrometry‐based phosphoproteomics survey	**Silmitasertib**, *abemaciclib, dinaciclib*	Inhibition of CDKs, display the significant activity of antiviral, decrease the cytopathic effect	^20^, ^30^, ^122^, ^277^, ^278^, ^279^, ^280^
Casein kinase 2	Sequence analyses and data from phosphorylation studies	**CIGB‐325, silmitasertib,** *quercetin, enzymatically modified isoquercitrin (EMIQ)*	CK2 inhibitors, alleviate the symptoms, potent activity in antiviral	^125^, ^281^, ^282^, ^283^
p38 MAPK	Drug repurposing strategy with screening of approved drugs	*SB203580, pamapimod, ralimetinib, MARK13‐IN‐1, ARRY797 and 20(S)Ginsenoside*	Reduce replication of SARS‐CoV‐2, decrease the mRNA of cytokines	^112^, ^284^
JAK–STAT	Screening the US FDA‐approved JAK inhibitors	**Ruxolitinib, baricitinib, tofacitinib, ivermectin,** *fedratinib*	Suppress cytokine storm and endocytosis	^285^, ^286^, ^287^, ^288^, ^289^, ^290^, ^291^, ^292^, ^293^
GSK‐3	Screening the licensed drugs for other diseases	*Iithium, Iithium chloride (LiCl), kenpaullone, tideglusib, thiadiazolidinone*	GSK‐3 targeting inhibitor, restrain the activity of SARS‐CoV‐2 Mpro	^133^, ^136^, ^234^
IL‐6	Repurposing from a using in the treatment of cytokine storms caused by chimeric antigen receptor T cell (CART) therapy	**Siltuximab, tocilizumab, sarilumab**	IL‐6 inhibitors, decrease cytokine levels, ameliorate the symptoms of systemic toxicity	^139^, ^140^, ^141^, ^142^, ^143^, ^144^
IL‐1β	Repurposing from a using in the treatment of inflammatory disorders and rheumatoid arthritis	**Canakinumab, anakinra**	IL‐1β antagonist and IL‐1 receptor antagonist	^145^, ^146^, ^147^, ^148^, ^149^
IFN‐γ	Drug approved for primary hemophagocytic lymphohistiocytosis (HLH)	**Emapalumab**	IFN‐γ monoclonal antibody, alleviate hyperinflammation, and improve respiratory conditions	^150^, ^151^
TNFα	Repurposing from chronic inflammatory diseases, by high‐throughput screening technology	**Infliximab and adalimumab**	Mitigate or ameliorate the COVID‐19 disease course	^152^, ^153^, ^154^, ^155^
IL‐12/23	Repurposing from chronic inflammatory and autoimmune diseases	**Risankizumab, guselkumab, tildrakizumab, ustekinumab**	Inhibit IL‐12/23	^156^, ^157^, ^158^, ^159^, ^160^, ^161^, ^162^
IL‐17A	Screening the inhibitors of IL‐17A	**Secukinumab, ixekizumab**	Relieve the symptoms or asymptomatic of COVID‐19	^163^, ^164^
GM‐CSF	Repurposing from autoimmune diseases and inflammatory diseases,	**Sargramostim, molgramostim, mavrilimumab, lenzilumab, otilimab, gimsilumab, TJ003234**	Human recombinant GM‐CSF, GM‐CSF antibodies, GM‐CSF inhibitors	
BRD2	Repurposing drugs for HIV	*JQ1 and ABBV‐744*	BET inhibitors, decrease the levels of ACE2 mRNA	^167^
Thymosin	Repurposing from the treatment of chronic inflammation and autoimmune diseases	**Thymosin alpha 1 (Tα1)**	Be useful in contributing to the reconstruction of effective T‐cell immunity, potentially inhibit cytokine storms	^173^, ^174^
Immunoglobulin	Repurposing from the treatment of chronic inflammation and autoimmune diseases	**Immunoglobulin**	Regulate cytokine responses and immune cell functions	^175^, ^176^
PI3K/Akt/mTOR	Repurposing inhibitors of PI3K/Akt/mTOR	**Ebastine, sirolimus, everolimus, temsirolimus, tacrolimus,** *idelalisib, triciribine, MK‐2206*	Exert downregulation of excessive inflammatory reactions	^178^, ^180^, ^181^
NF‐κB	Repurposing of inhibitors targeting NF‐κB	**Hydroxychloroquine, macrolide antibiotics, dexamethasone, N‐acetylcysteine,** *phillyrin (KD‐1), pyrazole derivative*	Inhibit NF‐κB, anti‐inflammatory agents	^183^, ^184^, ^185^, ^186^
p38 MAPK	Repurposing of inhibitors targeting p38	**Losmapimod, hydroxychloroquine, silymarin**	Inhibit p38, reduce the inflammatory responses	^182^, ^187^
JAK/STAT	The downstream of IL‐6 and other various cytokines involved in the CS	**Baricitinib, ruxolitinib, tofacitinib**	Improve the inflammatory condition in SARS‐CoV‐2‐infection and limit lung pathology	^130^, ^188^, ^189^, ^190^
GSK‐3	Repurposing from targeting the type 2 diabetes or neurodegenerative and psychiatric disorders	*LY2090314, AZD‐1080*, and *COB‐187*	Attenuate spike protein‐induced chemokine CXCL10 expression	^191^, ^192^, ^193^, ^194^, ^195^

^1−3^ *Note*: Repurposed drugs have been confirmed in cell culture (*italic*), animal model (underline), and human clinical trials (**in bold**).

The advent of Spike protein‐based vaccines has abated the global burden; however, they were not found to be effective against all the variants of concern due to the rapid evolution of SARS‐CoV‐2.^8^, ^9^ In an effort to restrict the opportunities for SARS‐CoV‐2 to spread and mutate, there has been great advocacy for immunization, because the higher the proportion of a population vaccinated, the lower the number of susceptible individuals.^9^ In addition to vaccines, there has been a shift to focusing on the development of small molecules targeting the critical targets that are more conserved among the viral replication machinery. Such targets would be expected to serve as potentially effective therapeutic approaches for immunosuppressed individuals who are unable to mount responses even after two vaccine doses.^8^, ^10^

### 3.1 Repurposed drugs targeting the viral proteins associated with the SARS‐CoV‐2 viral life cycle

SARS‐CoV‐2 uses the spike protein to recognize human cells and induce the fusion of the viral and human cell membranes through receptor binding.^28^, ^30^, ^294^ These high‐affinity interactions are essential for viral entry and are therefore key targets in the treatment of COVID‐19. After priming by TMPRSS2 or Furin,^56^, ^256^ the virus enters the cytoplasm through ACE2‐, AXL‐, or CD147‐mediated endocytosis.^13^, ^27^ The virus then releases the viral genetic material and replicates (via RdRp, 3CLpro, and PLpro).^52^ Accompanying stages of viral infection involve protein synthesis, assembly, viral particle release and infection of adjacent host cells. Repurposed approved/clinical drugs interfere with different viral targets and directly block virus infection through inhibiting specific steps of the viral infection cycle.^48^, ^58^, ^225^, ^233^

#### 3.1.1 Repurposed drugs targeting the Spike glycoprotein or affecting S‐host cell receptor binding

Viral replication and spread mainly relies on the interaction between the Spike protein and host cell surface receptors, thereby mediating membrane fusion and causing viral invasion. Some studies report that development of drugs, including antibodies or small molecular inhibitors that target the S protein or affect the binding of the S protein to the host cell receptor is beneficial for constraining coronavirus infection. Researchers repurposed the neutralizing antibodies from convalescent SARS patients and tested their ability to block SARS‐CoV‐2‐S‐driven entry; these antibodies efficiently offered at least partial protection against SARS‐CoV‐2.^25^ Through high‐throughput screening of a human scFv phage library from Geneservice using an S‐RBD protein as the target, researchers identified a specific antibody against the S‐RBD and blocked Sp‐ACE2 interaction effectively.^221^ Via a drug repurposing strategy based on screening of approved antiviral/antiparasitic/antiprotozoal drugs, mefloquine and its derivatives were identified; their mechanisms of action were revealed to be targeting the viral Spike protein or preventing Sp‐ACE2 binding, and in effect acting prophylactically as an entry inhibitor.^222^ Through repurposing of clinically approved drugs and Chinese herbal medicines, the previously anti‐inflammation/viral/cancer compounds, andrographolide and Quercetin, were discovered to bind well to both the Spike protein and ACE2.^223^ Traditional Chinese medicine has shown promise for COVID‐19. In a network pharmacology study that identified 112 active compounds in Ephedra‐Glycyrrhiza, the agents Kanzonol F, Gancaonin G, and Supraene were shown to exhibit a high binding affinity to the spike protein.^295^ This suggests potential efficacy against SARS‐CoV‐2. Another study reported identification of two new agents with different binding modes of action having higher antiviral potential, through screening of a panel of approved drugs in a cell model of SARS‐CoV‐2. The agents were the anti‐inflammatory drug cepharanthine (which inhibited SARS‐CoV‐2 entry through the blocking of viral binding to target cells), and the HIV protease inhibitor, nelfinavir (which suppressed viral replication partly by protease inhibition). Combined use of these drugs led to a synergistic effect that limited SARS‐CoV‐2 proliferation.^224^ In addition to this study, using a AlphaScreen‐based high‐throughput system, researchers from a different group found that ceftazidime inhibited SARS‐CoV‐2 infection in vitro by blocking Sp‐ACE2 interaction, through 3581 small compounds with known functions and structure from different databases. This demonstrates the advantages and reliability of drug repurposing (Figure 2).

#### 3.1.2 Repurposed drugs targeting viral entry receptors

The host receptors (ACE2, AXL, and CD147) contribute to cell entry of SARS‐CoV‐2 through binding with different domains of Spike protein.^26^, ^30^ Therefore, repurposed antibodies or small compounds targeting host receptors or blocking the interaction with S protein should be effective therapeutic strategies that may be used to treat COVID‐19. Recently, to achieve fast and reliable results, the in silico drug repurposing strategy combined with molecular docking was used in screening compatible inhibitors for novel COVID‐19 drug development. A high‐throughput virtual screening following with followed by molecular dynamics simulation studies identified ledipasvir, vancomycin from an US FDA‐approved drug library; drugs were screened for their ability to, through interfering block the binding of S protein to ACE2 or CD147, which are potential candidates for further investigation as possible treatments of COVID‐19 and novel drug development.^226^ In this study, estradiol benzoate showed the most favorable free binding energy with CD147, implicating suggesting that this drug may be a potentially candidates for further investigation as a treatment for s of COVID‐19.^226^ Using the similar approach, four previously approved compounds, ketazolam, methylnaltrexone, ethynodiol diacetate, and naloxone, were found to displayed better activity than the currently investigated drugs, HCQ, lopinavir, and remdesivir, for treatment of COVID‐19. Although they are multitargeted, these drugs could prevent viral entry into host cells by targeting ACE2.^227^ Through repurposing of clinically approved drugs and Chinese herbal medicines, other studies revealed that quercetin, andrographolide, glycyrrhizic acid, and luteolin are potential ACE2 inhibitors that may be efficacious against COVID‐19.^223^ Through network pharmacology and molecular docking methods, Howxiang Zhengqi Powder, Jinhua Qinggan Granules, Xuebijing injection^296^ were found to exhibit strong binding activity and potential to inhibit viral entry.

Bemcentinib was reported to be a highly selective and potent AXL inhibitor with antiviral activity and was specifically show to block entry of SARS‐CoV‐2 into pulmonary and bronchial epithelial cells.^259^ This drug was fast‐tracked as the first potential treatment for COVID‐19 and was entered into a randomized phase II trial as part of the Accelerating COVID‐19 Research & Development multicenter of United Kingdom.^70^ This assessment shields light on patients with thoracic cancers that were reported to have elevated expression of AXL and that were afflicted with COVID‐19.^70^ Combining the KINOMEscan‐LINCS biochemical kinase profiling database with the ChEMBL database of targets associated with SARS‐CoV, our colleagues identified gilteritinib,^12^ an approved drug for treatment of relapsed/refractory acute myeloid leukemia (AML); as an AXL inhibitor, the agent shows robust activity against different subvariants of concern in SARS‐CoV‐2 infection models. These findings are supported by another group's report that gilteritnib was successful in causing remission in a FLT3‐mutant‐positive AML patient with severe COVID‐19.^260^ These findings support further clinical investigation of gilteritinib as an antiviral agent and potential therapy for the symptoms of COVID‐19. Two other repurposed drugs, nintedanib and imatinib, which target AXL or AXL‐related kinases, have also shown potential therapeutic activity against COVID‐19 disease and now are in clinical trials.^12^

#### 3.1.3 Repurposed drugs targeting proteases for priming

SARS‐CoV‐2 was reported to enter cells via binding to host cell receptors, followed by priming by TMPRSS2, Furin and CatL. N‐0385, a peptidomimetic tetrapeptide compound identified in a small library of drugs screened against influenza A virus, was reported to be a TMPRSS2 inhibitor, which impairs the proteolytic activation of Spike protein of different SARS‐CoV‐2 subvariant viruses of concern and blocks viral‐cell membrane fusion and entry.^58^ Camostat mesylate, a clinically proven serine protease inhibitor that was reported to be active against TMPRSS2 in SARS‐CoV, shows robust blocking of SARS‐Cov‐2‐S entry into host cells. Combination of camostat mesylate with E‐64d, an inhibitor of CatB/L, led to full inhibition of virus entry, suggesting a treatment option for SARS‐CoV‐2.^25^, ^255^ Other inhibitors, including nafamostat and aerosolized aprotinin, have been shown to attenuate TMPRSS2 protease activity; however, only camostat has been a candidate for investigation in a phase 2 clinical trial.^256^ Further studies are needed for investigation of the therapeutic potential of these agents as therapeutics for COVID‐19.

The subtilisin‐like proprotein convertase, Furin, is another attractive target and its inhibitors have shown activity against multiple furin‐dependent pathogens by twofold, including Ebola virus and bird flu virus (A H5N1), via restraining viral entry and enhancing the immune response for viral clearance.^33^ Furin has recently been shown to cleave the SARS‐CoV‐2 S protein, and thus Furin inhibitors could potentially be repurposed as novel drugs for COVID‐19.^297^, ^298^ Through structure‐based virtual screening and post‐screening biochemical assays, it was found that diminazene can effectively inhibit the activity of Furin.^257^ However, there are currently no ongoing clinical trials investigating direct small molecule inhibitors of Furin for COVID‐19.^56^

#### 3.1.4 Repurposed drugs targeting RdRp

The in silico drug repurposing strategy has also been used for screening of RdRp inhibitors screening. Vancomycin were recently identified as drugs targeting RdRp and blocking virus replication recently.^226^ Remdesivir showed favorable binding energy with RdRp and other targets important for virus,^227^ and favipiravir causes lethal mutagenesis upon incorporation into the virus RNA without causing cytotoxicity in mammalian cells; this agent has been reported to effectively inhibit SARS‐CoV‐2 infection in clinical trials.^228^, ^229^, ^230^, ^231^ Molnupiravir (MK‐4482, EIDD‐2801) is another antiviral drug repurposed as a therapeutic agent for the management of COVID‐19; both molnupiravir and favipiravir were approved by the US FDA.^225^ Other compounds that bind the SARS‐CoV‐2 RdRp, including galidesivir, ketazolam, was identified through computational studies and high‐throughput screening of US FDA‐approved drugs.^227^, ^299^ Galidesivir binds at the noncatalytic location and induces a conformational change in RNA polymerase, and the other compounds show low binding energy to RdRp. Moreover, a molecular docking study confirmed the binding of remdesivir and galidesivir to RdRp, and identified novel drugs, including ribavirin, sofosbuvir, and tenofovir, as candidates for newly emerged coronaviruses.^232^ To understand whether the individual agents or combinational therapies are efficacious against SARS‐CoV‐2 infection, further investigations are recommended through preclinical and clinical studies.^227^, ^232^, ^299^

Screening studies involving traditional Chinese medicine compounds identified theaflavin as a potential SARS‐CoV‐2 RdRp inhibitor. Similar compound structures were identified through a molecular docking study as able to target RdRp of SARS‐CoV‐2, SARS‐CoV, and MERS‐CoV.^300^ With a broad spectrum of antiviral properties, andrographolide, the main active component isolated from the extract of the herb *Andrographis paniculate*, inhibited infection by various viruses including HIV^301^ and influenza A virus.^302^ Andrographolide was demonstrated to bind well to RdRp, which suggests that it may be active against SARS‐CoV‐2.^248^ In addition, the binding effect of patchouli alcohol to RdRp was demonstrated.^248^ Patchouli alcohol, a tricyclic sesquiterpene compound extracted from the traditional Chinese medicine *patchouli*, has biological effects including antiviral, immunomodulatory, and antitumor.^303^

#### 3.1.5 Repurposed drugs targeting viral proteases

Previous research has demonstrated that two SARS‐CoV‐2 proteases, Mpro and PLpro, are relatively stable compared with the Spike protein.^234^ Therefore, they are attractive targets for antiviral drug development and for developing anti‐SARS‐CoV‐2 therapies.^234^ Mpro inhibitors exert their inhibitory effect by covalently or noncovalently modifying the catalytic Cys145 of Mpro.^304^ Through fluorescence resonance energy transfer (FRET)‐based enzymatic assays for SARS‐CoV‐2 Mpro, and applying this technique toward screening a specific library of protease inhibitors, boceprevir, GC‐376, and calpain inhibitors II and XII were identified to have potent activity against anti‐SARS‐CoV‐2.^305^, ^306^ Using a similar method, baicalin and baicalein were shown to be noncovalent inhibitors of SARS‐CoV‐2 Mpro, with high ligand binding efficiency due to their unique binding mode.^235^ In another study, the crystal structures and NMR analysis revealed the parent compound of GC‐376, GC‐373, to covalently bind to SARS‐CoV‐2 Mpro.^236^ The X‐ray crystal structure of Mpro in complex with carmofur showed that carmofur can be bound to catalytic Cys145 of SARS‐CoV‐2 Mpro. Thus, this is a promising compound and further studies are warranted.^237^ Through a combination of structure‐based virtual and high‐throughput screening, Ebselen (an antioxidant drug) was identified from more than 10,000 compounds as showing antiviral activity in cell‐based assays.^234^ Using combined computational screening coupled with molecular docking studies, another group discovered that luteolin and CQ bind to SARS‐CoV‐2 Mpro, suggesting potential antiviral molecules. However, molecular dynamics simulations were not carried out.^238^ Noticeably, seven isolated tanshinones derived from *Salvia miltiorrhiza* (Danshen), including tanshinone IIA, tanshinone IIB, methyl tanshinonate, cryptotanshinone, tanshinone I, dihydrotanshinone I, and rosmariquinone, strongly inhibited PLpro.^307^ In addition, hirsutenone,^308^ xanthoangelol E,^308^ isobavachalcone, 4′‐*O*‐methylbavachalcone, psoralidin,^309^ and tomentin A‐E^310^ target PLpro.

A novel in‐cell protease assay (ICP) combined with docking studies were used with a library of 64 repurposed drugs and led to identification of several SARS‐CoV‐2 Mpro inhibitors, including MG‐101, lycorine HCl, nelfinavir mesylate, lomibuvir, and BMS‐707035.^239^ Using molecular modeling and quantum chemical methods, another group interrogated the activity of lopinavir and ritonavir on SARS‐CoV‐2 Mpro. Moreover, lopinavir has been investigated in a Phase IV clinical trial.^240^, ^241^, ^242^ Ensitrelvir is a novel oral SARS‐CoV‐2 M pro inhibitor that is being investigated in Phase II and III clinical trials.^243^, ^244^ PF‐07321332 (PAXLOVID™) has been approved by US FDA to treat SARS‐CoV‐2 as novel COVID‐19 oral antiviral regent. Recently, ensitrelvir was demonstrated to show antiviral activity against the Omicron variant in an animal study, although clinical studies have not yet been carried out.^243^ In a library screening of 1900 clinically safe drugs for antiviral activity against SARS‐CoV‐2, Masitinib was identified as exhibiting the highest potency against 3CLpro, and X‐ray crystallography and biochemistry showed it is a competitive inhibitor of 3CLpro.^233^ Masitinib has been under investigation in a Phase II clinical trial.^233^ The ethanol extract of *S. baicalensis* and its ingredients, baicalein, were found to inhibit the replication of SARS‐CoV‐2 via targeting 3CLpro.^311^ In addition, EGCG and theaflavin, the main active ingredients of green tea and black tea, exhibited inhibitory activity against 3CLpro in a dose‐dependent manner.^312^ Similar studies, combined with molecular dynamics simulations, led to identification of diosmin (NCT04452799), selinexor (NCT04349098), ivermectin (NCT04668469), and elbasvir as showing a high affinity for Mpro; diosmin mixture with hesperidin and selinexor is under clinical investigation against SARS‐CoV‐2.^245^ In addition, nelfinavir was identified as interacting with SARS‐CoV‐2 Mpro, and the combination of cepharanthine with nelfinavir showed synergistic potential against symptoms of COVID‐19.^224^

PLpro inhibiters are able to block viral replication by shutting down the active site of PLpro, and they are believed to decrease the activity of PLpro by binding at an allosteric site.^263^ Noncovalent PLpro inhibitors with favorable pharmacokinetic properties and the first‐in‐class covalent PLpro inhibitors have been designed.^313^ GRL0617, developed by Ratia et al., was originally used as an inhibitor of SARS‐CoV‐1 PLpro and recently was investigated as an inhibitor of SARS‐CoV‐2 PLpro.^314^ A drug‐repurposing screening, carried out using a MedChemExpress bioactive compound library, identified tropifexor as a novel and promising PLpro inhibitor against SARS‐CoV‐2.^315^ Recently, through virtual screening of the US FDA‐approved drug library, nine compounds were identified and in vitro biochemical and cell culture‐based studies revealed anti‐SARS‐CoV‐2 PLpro activity of mefloquine and lopinavir.^249^ The cocrystal structure of SARS‐CoV‐2 PLpro and GRL0617 was also reported, and GRL0617 was found to be a noncovalent inhibitor binding to the ubiquitin‐specific proteases domain of PLpro with antiviral effects in vitro.^250^ The use of ICP and docking studies, combined with screening of a library of 64 repurposed drugs, led to identification of sitagliptin and daclatasvir HCl as binding to PLpro and blocking the entrance to the catalytic site.^239^ Computer docking screening and molecular dynamics simulation studies suggested interaction between flavonoids, such as quercetin, or their derivatives, and the binding pocket of SARS‐CoV‐2 PLpro and Mpro.^251^ In another study, molecular docking results showed that andrographolide and baicalin bound well to PLpro and Mpro, suggesting that these agents have potential anti‐SARS‐CoV‐2 activity.^248^ Use of a new chemical approach, called HyCoSuL (Hybrid Combinatorial Substrate Library), led to development of two irreversible inhibitors targeting SARS‐CoV‐2‐PLpro, VIR250 and VIR251, both exhibiting potent and selective inhibitory activity against SARS‐CoV‐2 PLpro.^252^ Plaque reduction assays revealed naphthalene‐based derivatives and PLpro inhibitors designed for SARS‐CoV are able to inhibit SARS‐CoV‐2 PLpro^253^ (Figure 2). However, up to now, no drugs targeting PLpro have been recruited in clinical trials, and further preclinical and clinical studies are necessary.

### 3.2 Repurposed drugs targeting the host protein kinases

Host kinases are essential for all stages of the virus life cycle and are linked to the transmission of SARS‐CoV‐2 and symptoms related to SARS‐CoV‐2 infection, including cytokine synthesis, inflammation, and fibrosis. These potential effects of kinases make them attractive targets in the battle against infection or pandemic.xs^83^, ^84^, ^93^, ^94^, ^112^, ^129^, ^130^, ^133^ Many kinases can be targeted by inhibitors, and these promising kinase inhibitors may be beneficial in severe COVID‐19 cases through preventing infection by directly targeting the virus and reducing clinical symptoms. Moreover, kinase inhibitors can reduce cytokine production via affecting related pathways and boosting the efficacy of other antiviral treatments for SARS‐CoV‐2. Numerous kinase inhibitors are being evaluated in clinical studies involving patients with coronavirus infections.^14^, ^74^, ^131^, ^177^, ^178^, ^182^, ^188^, ^202^ Here, we discuss a panel of repurposed kinase inhibitors that reduce symptoms and enhance patient outcomes (Figure 3).

#### 3.2.1 Repurposed drugs targeting SRC and NAK

SRC family kinases have been reported to be crucial for the viral life cycle in MERS‐CoV and West Nile virus and may be potential targets for SARS‐CoV‐2.^83^, ^84^ An inhibitor of SRC/ABL kinases, saracatinib, is able to restrain the life cycle of MERS‐CoV using concentrations in the micromolar range.^83^ Moreover, in combination with gemcitabine, a thymidylate synthase inhibitor, the anti‐MER‐CoV efficacy of saracatinib was further strengthened.^261^ In addition to saracatinib, dasatinib is also able to suppress the assembly of dengue virus by targeting SRC family kinases, particularly c‐Src and Fyn.^262^ Although saracatinib, dasatinib and gemcitabine exhibit inhibitory effects on viral proliferation, more investigation into their antiviral efficacy with respect to SARS‐CoV‐2 is needed.

As the NAK family is regarded as an essential mediator of viral infection, assembly and egress for SARS‐CoV‐2, NAK could be an appropriate candidate for antiviral strategies against SARS‐CoV‐2 infection. Therefore, numerous drugs that have been authorized by the US FDA^316^ for treatments of other diseases are repurposed and investigated in research against SARS‐CoV‐2 by targeting NAK. Sunitinib, a multitargeted kinase inhibitor with targets including AAK1 and GAK, and erlotinib, an EGFR tyrosine kinase inhibitor, both show activity against dengue, Ebola and HCV viruses by blocking their entry, assembly, and traffic.^69^, ^264^, ^265^, ^266^ One study showed that baricitinib, a JAK inhibitor licensed by the US FDA for its anti‐inflammatory activity, is a prospective alternative to treat COVID‐19.^263^ It was reported that the US FDA‐approved baricitinib to treat patients in combination with remdesivir. Moreover, baricitinib mitigated ARDS effectively in critically ill patients with COVID‐19.^263^ Therefore, the migration and intracellular assembly of SARS‐CoV‐2 were inhibited by treatment with baricitinib in patents. However, the clinical effectiveness of baricitinib resulting from NAK‐targeting is controversial.^88^ Therefore, further clinical studies on the targeted effects of baricitinib in patients are warranted.

#### 3.2.2 Repurposed drugs targeting EGFR

EGFR overexpression not only influences cell proliferation but also affects inflammation, immune thrombosis, and fibrosis, which eventually leads to serious and potentially deadly disease in patients diagnosed with COVID‐19. Gefitinib and erlotinib, the EGFR‐targeting inhibitors, have been verified to be active against HCMV and HCV both in vitro and in vivo.^267^, ^268^, ^269^ These agents are currently repurposed for COVID‐19 treatment according to their potential effectiveness against SARS‐CoV‐2. Osimertinib, a potent inhibitor of EGFR, authorized for non‐small cell lung carcinoma treatment, has demonstrated anti‐SARS‐CoV‐2 activity in in vitro studies through suppressing S protein.^122^ However, cytotoxicity may be a limiting factor for this agent. Nimotuzumab, an anti‐EGFR antibody, reduced inflammation and fibrosis in COVID‐19 patients with moderate and severe disease.^93^ Due to the small sample size in this study, however, further studies will need to be carried out in a larger cohort of patients.

#### 3.2.3 Repurposed drugs targeting PI3K/Akt/mTOR

Inhibitors of the PI3K/Akt/mTOR pathway can exert antiviral effects. For example, the mTOR inhibitors, everolimus and rapamycin, have been shown to suppress HIV or MERS infection in preclinical settings.^105^, ^317^ Recent reports suggest a role for the PI3K/Akt/mTOR pathway in anti‐SARS‐CoV‐2. Repurposing of targeted inhibitors of this pathway could help downregulate excessive inflammatory responses and exhibit antiviral effects. There are numerous inhibitors targeting PI3K/Akt/mTOR that could potentially exert potent effects.^107^, ^178^, ^179^

Pictilisib, an effective anti‐PI3K inhibitor, was shown to reduce viral replication in cells with an IC50 of 2.58 μΜ.^292^ Further, VPS34‐IN1, an inhibitor of the class III PI3K Vps34, significantly suppressed SARS‐CoV‐2 replication in vitro.^271^ The Akt inhibitor, MK‐2206, exhibited significant antiviral effects against SARS‐CoV‐2 in vitro, likely via induction of enhanced autophagy, secondary to the stabilization of Beclin‐1.^106^ In cells infected with MERS, the mTOR inhibitor, rapamycin, was shown to block MERS infection.^105^ One case report showing the positive effect of rapamycin in COVID‐19 patients has attracted attention.^179^ There are also other inhibitors of mTOR, including metformin, tacrolimus.^272^, ^273^, ^274^ Tacrolimus can be potentially effective owing to its modulation of cytokines associated with T cells.^273^ Omipalisib and SF2523, two dual mTOR and PI3K inhibitors, efficiently blocked SARS‐CoV‐2 replication.^275^, ^276^ In addition, the dual RAF and MEK inhibitors, sorafenib inhibited viral infection and replication.^318^ The RAS inhibitor, lonafarnib, blocked SARS‐CoV‐2 replication with IC50 4.99 μM.

#### 3.2.4 Repurposed drugs targeting CDKs and CK2

CDKs are involved in cell proliferation and play a role in viral infectivity. Up to now, numerous inhibitors targeting CDKs have been developed or approved for cancer treatment. Moreover, many have been proposed to be repurposed for combating SARS‐CoV‐2. Silmitasertib, a CDK inhibitor in clinical trials for treatment of COVID‐19, displayed significant antiviral activity with an IC50 of 1.28 M.^20^ Further, it has been reported that abemaciclib, an inhibitor targeting CDK4/6, and dinaciclib, an anti‐CDK1/2/5/9 inhibitor, were able to suppress the activity of SARS‐CoV‐2 infection.^122^ As a drug approved for breast cancer therapy, abemaciclib was able to decrease the cytopathic effect (CPE) of SARS‐CoV‐2 by 40%.^30^ Other inhibitors of CDKs, such as flavopiridol, roscovitine, palbociclib, and ribociclib, exhibited activity against HIV, HCMV, HSV.^277^, ^278^, ^279^, ^280^ The repurposing of these diverse inhibitors may represent a novel effective therapeutic strategy to fight SARS‐CoV‐2 infection.

In a recent study, CIGB‐325 was investigated for its role in coronavirus infection based on targeting CK2.^281^ Half of randomly selected patients afflicted with COVID‐19 were given CIGB‐325 (2.5 mg/kg for 5 days) along with standard care, the other half were given standard care only.^281^ Chest computed tomography was improved in patients receiving the CK2 inhibitor.^281^ Through computational analysis, the mechanism whereby CIGB‐325 alleviates COVID‐19 symptoms was elucidated.^282^ However, due to the small scale of the study, more investigation is warranted. Silmitasertib, an inhibitor of CK2 under clinical investigation for cholangiocarcinoma, showed potent activity in antiviral assays and thus has entered clinical studies for COVID‐19. Other CK2 inhibitors, including quercetin and enzymatically modified isoquercitrin (EMIQ), are also promising candidates for COVID‐19 treatment.^125^, ^283^

#### 3.2.5 Repurposed drugs targeting p38 MAPK

Signaling via p38 has recently been proven as one of the key pathways for the replication of SARS‐CoV‐2. p38 MAPK regulates not only viral infection, but also inflammatory responses. Hence, numerous drugs that have been approved are considered appropriate options for the treatment of COVID‐19. Following treatment with SB203580, a p38 inhibitor, the replication of SARS‐CoV‐2 was reduced to a large extent without obvious cellular cytotoxicity.^112^ This was further verified by an anti‐SARS‐CoV‐2 N protein antibody‐based assay.^112^ Moreover, the mRNA of cytokines that are increased during infection, such as IL‐6, TNF, and CCL20, were also decreased by SB203580.^112^ Recently, a group reported that pamapimod, a selective inhibitor of p38 MAPKα, evaluated clinically for the treatment of rheumatoid arthritis (RA), effectively suppressed replication of SARS‐CoV‐2 in vitro. The similar antiviral potency against different emerging variants of concern, including alpha, beta, gamma, delta, and omicron, is remarkable.^284^ Other inhibitors of MAPK, such as ralimetinib, MARK13‐IN‐1, ARRY797, and 20(S)Ginsenoside, have shown antiviral activity or anti‐inflammatory efficacy on other viruses.^112^ The potential of these agents as treatments for SARS‐CoV‐2 warrants investigation.

#### 3.2.6 Repurposed drugs targeting JAK–STAT

Currently, repurposing of inhibitors of JAK/STAT as a treatment for cytokine storm associated with COVID‐19 is under investigation. Ruxolitinib, an orally available JAK inhibitor, has shown significant reduction in the levels of increased cytokine production linked to hyperimmune syndrome.^285^ The efficacy and function of ruxolitinib was evaluated in COVID‐19 patients, and ruxolitinib was found to have potential in mitigating ARD.^286^, ^287^ A recent study demonstrated the JAK1/2 inhibitor, baricitinib, as being an effective agent for COVID‐19 with minimal side effects.^288^ Another report suggested the release of cytokines and endocytosis associated with SARS‐CoV‐2 infection could be suppressed by baricitinib.^289^ Tofacitinib, a treatment for RA, is an orally available JAK inhibitor with demonstrated activity against COVID‐19.^290^, ^291^ The targeted JAK2 inhibitor, fedratinib, was found to be beneficial to COVID‐19 patients suffering from cytokine storm due to its inhibition of TH17‐related cytokines.^292^ A broad‐spectrum drug, ivermectin, was found to inhibit SARS‐CoV‐2 with an IC50 2.0 μM through blocking the Stat3 signaling pathway and has been proposed to be a potential candidate for the management of COVID‐19.^293^ However, side effects of JAK/STAT inhibitors, such as ruxolitinib, remain controversial and further investigations are required.

#### 3.2.7 Repurposed drugs targeting GSK‐3

Given the remarkable impact of GSK‐3 on physiological processes and vital roles in the onset of clinical symptoms related to viral infections, GSK‐3 is considered a promising target of SARS‐CoV‐2. Indeed, a large number of inhibitors have been repurposed and are under investigation, including lithium, lithium chloride (LiCl), kenpaullone, tideglusib, and thiadiazolidinone. Lithium is a GSK‐3‐targeting inhibitor that was approved for patients diagnosed with bipolar disorder.^136^ Treatment with LiCl and kenpaullone led to reduction of titer in SARS‐CoV‐1.^133^ According to a recent report, tideglusib, designed to treat Alzheimer's disease, showed activity against SARS‐CoV‐2 Mpro with IC50 of 1.5 μM.^234^ Another study demonstrated potent antiviral activity of thiadiazolidinone against COVID‐19^234^ (Figure 3).

### 3.3 Repurposed drugs targeting immunoregulators

The SARS‐CoV‐2 virus can cause clinical symptoms ranging from mild, such as fever and cough, to moderate, such as pneumonia and localized inflammation that require hospitalization, to severe symptoms, such as ARDS, cytokine storm, multiorgan failure, and fatality.^5^, ^6^, ^22^, ^24^ There is considerable evidence that severe medical complications, such as immunopathological damage, with highly elevated levels of proinflammatory cytokines, may be responsible for the poor outcomes associated with COVID‐19.^23^ Currently, there are no specific treatments for severe complications, such as cytokine storm induced by SARSCoV2. However, several small molecule drugs and repurposed therapies are currently under investigation^23^, ^138^, ^139^ (Figure 4).

#### 3.3.1 Repurposed antibodies or drugs targeting proinflammatory cytokines or receptors

Excessive secretion of IL‐6 leads to tissue damage and impairs NK cell antiviral activity.^138^, ^139^ Therefore, targeting of IL‐6 or IL‐6R could be beneficial for SARSCoV2 infected patients. A number of preclinical and clinical studies have confirmed the safety and efficacy of the IL6 antibody, siltuximab, and the IL6R antibodies, tocilizumab and sarilumab, showed promise in decreasing cytokine levels, ameliorating symptoms of systemic toxicity, and reducing the requirements for adjuvant and other therapies, including vasoactive drugs, glucocorticoids and respiratory support.^139^, ^140^, ^141^, ^142^ There are presently more than 30 trials investigating tocilizumab for treatment of SARS‐CoV‐2. Tocilizumab has been used for treatment of cytokine storms.^143^ Tocilizumab is able to attenuate the levels of IFN‐γ, IL10, and IL2, and treat refractory hemophagocytic lymphohistiocytosis (HLH) with expansion of cytotoxic T and NK cells.^144^

Monoclonal antibodies blocking IL‐1β, IFN‐γ, TNF‐α, IL‐12/23, IL‐17A, and GM‐CSF have been repurposed for SARS‐CoV‐2‐induced cytokine storm immune disruption. Several drugs targeting IL‐1β signaling have been repurposed for clinical treatment of COVID‐19, including the IL‐1β antagonist, canakinumab (Novartis, developed for suppression of inflammation in patients with disorders of autoimmune origin), and the IL‐1 receptor antagonist anakinra (Kineret, approved for the treatment of RA in 2001).^145^, ^146^, ^147^, ^148^ There are currently over 30 clinical trials, investigating single agent or combination treatment, registered in ClinicalTrials.gov.^148^, ^149^ Emapalumab (Gamifant), an IFN‐γ monoclonal antibody approved for primary HLH, has been repurposed as a potential therapeutic for COVID‐induced cytokine storm, considering the contribution of IFN‐γ to cytokine storm as mentioned above.^150^, ^151^ A randomized clinical trial (Italy (NCT04324021) was carried out to investigate the efficacy of emapalumab on alleviating hyperinflammation and improving respiratory conditions, in combination with anakinra. However, this investigation was eventually terminated, suggesting additional studies are needed to explore the potential of blocking IFN‐γ in COVID‐19.

Blocking of TNFα is supported by numerous studies as a potential treatment strategy for excessive cytokine release and hyperinflammation associated with COVID‐19. The approved TNFα antibodies, infliximab and adalimumab, were originally indicated for chronic inflammatory diseases and are now considered for COVID‐19 treatment.^152^, ^153^, ^154^ Currently, there are four clinical trials investigating infliximab (NCT04425538, NCT04734678, NCT04593940, NCT04344249), and two clinical trials (ChiCTR2000030089, NCT04705844) evaluating the therapeutic potential of adalimumab against COVID‐19 according to ClinicalTrials.gov. In addition, through high‐throughput screening technology, researchers evaluated the effects of a library of 900 herbs/traditional Chinese medicine for effects on cytokine storm. Several herbs were found to target IL‐6 or the TNF‐α pathway, and some drugs were observed to inhibit both pathways.^155^ IL‐12/23 inhibitors, including risankizumab, guselkumab, tildrakizumab, and ustekinumab, were originally indicated for chronic inflammatory and autoimmune diseases, such as psoriasis and inflammatory bowel disease,^156^, ^157^, ^158^, ^159^ and are now under investigation as COVID‐19 therapies. The clinical efficacy of these inhibitors in clinical patients has been observed according to numerous reports.^160^, ^161^, ^162^ Currently, a randomized clinical trial is underway to evaluate the efficacy of risankizumab alone or in combination with remdesivir in COVID‐19 (NCT04583956). Blockade of IL‐17A is another potential option for coronavirus cytokine storm treatment. Several case reports have shown that the IL‐17A antagonists, secukinumab and ixekizumab, relieve the symptoms of COVID‐19 in patients with psoriasis, suggesting blocking of IL17A as a potential strategy for treatment of COVID‐19.^163^, ^164^ Moreover, results of a clinical trial carried out in northern Italy on COVID‐19 patients with psoriasis and treated with IL‐17A inhibitors showed a relatively low hospitalization rate (four out of 5206), suggesting a protective role of IL‐17A inhibitors against COVID‐induced cytokine storm. However, the reliability of this trial was compromised by a lack of a control population.^165^ Other clinical trials investigating ixekizumab (NCT04724629) and secukinumab (NCT04403243) as therapeutics for COVID‐19 are currently underway. In addition, the combination IL‐17A with IL‐6/IL‐6R inhibitors has been shown to have potential therapeutic effects in the context of COVID‐19 disease.^166^

It is predicted that the active ingredients of Maxing Ganshi decoction, kaempferol and quercetin, may inhibit the coagulation pathway triggered by IL‐6, as well as reduce the level of TNF‐α and IL‐1β.^319^ Glycyrrhizic acid can significantly reduce IL‐6 release from macrophages through the TLR pathway.^320^ Lutein, licoisoflavone B, fisetin, quercetin, glyasperin F, isolicoflavonol, and semilicoisoflavone‐B reduce NF‐κB, leukocyte migration, and inflammation.^321^ In addition to inhibiting viral replication, Lianhua Qingwen capsule and Liu Shen capsule^127^ were found to reduce proinflammatory cytokine production, including TNF‐*α*, IL‐6, and MCP‐1.

GM‐CSF has been shown to activate innate and adaptive immune responses, and it improves the ability of the body to fight against viruses. Human recombinant GM‐CSF, including sargramostim and molgramostim, are under consideration for the treatment of COVID‐19. SARS‑CoV‑2 infects epithelial cells and destroys the pulmonary physiological barriers; this leads to imbalanced GM‑CSF regulation and thus elevated G‑CSF levels in the peripheral blood of patients with COVID‑19. The blockade of GMCSF could be a viable treatment strategy for COVID19 to achieve clinical benefit. Notably, at least 10 clinical trials have been initiated using this strategy. GM‐CSF antibodies, such as mavrilimumab and lenzilumab, are indicated for autoimmune diseases and inflammatory diseases, such as RA or giant cell arteritis; these are now under investigation as potential therapies for COVID‐19. There are several clinical studies underway evaluating the effectiveness of mavrilimumab for severe COVID‐19 (NCT04447469, NCT04397497, NCT04492514, NCT04399980, NCT04463004). Similarly, clinical investigation of lenzilumab for severe COVID‐19 showed significant and rapid clinical improvement, reduction of inflammation, and reduced progression to ARDS (NCT04583969, NCT04351152, NCT04534725). In addition, several GM‐CSF inhibitors, including otilimab, gimsilumab, and TJ003234, are under evaluation in clinical trials for COVID‐19 (NCT04351243, NCT04376684, NCT04341116).

Other options include blockade of the transcriptional regulator BRD2, a host protein that controls transcription of IFN response in virus infection. A recent study showed that blocking BRD2 may restrain the cytokine storm response associated with COVID‐19.^167^ JQ1, a BET inhibitor, has been shown to reactivate latent HIV, which helps to eradicate the virus.^168^ Recently, with the treatment of JQ1 or ABBV‐744, levels of ACE2 mRNA decreased roughly twofold without toxicity to primary human bronchial epithelial cells.^167^ This suggests that JQ1 or ABBV‐744 may be a potential therapy for COVID‐19.

Blockade of thymosin is reported as an immune enhancer and widely used in the adjuvant treatment of chronic inflammation and autoimmune diseases.^169^, ^170^ Currently, thymosin was proposed to be useful in contributing to the reconstruction of effective T‐cell immunity in patients with COVID19, thereby potentially inhibiting cytokine storms.^171^ With an emergency use of thymosin, the symptoms and lung imaging improved significantly in a COVID‐19 patient.^172^ It has been reported that thymosin alpha 1 (Tα1) effectively reduces mortality associated with severe COVID‐19, however the effect of Tα inn restoring T Lymphocyte counts is controversial.^173^, ^174^ The safety of Tα1 in the treatment of COVID‑19 was also investigated in clinical trials (NCT04487444, NCT04428008). Another option is immunoglobulin, which regulates cytokine responses and immune cell functions and has been reported to show clinical benefit in severe sepsis patients.^175^ Notably, a case report of three patients revealed that a high‑dose of intravenous immunoglobulin in the early stages of clinical deterioration is able to prevent ARDS progression and improve the prognosis of COVID‑19.^176^

#### 3.3.2 Repurposed drugs targeting related signal pathways

The PI3K/Akt/mTOR pathway is involved in initiating the inflammatory response and producing cytokines, thus triggering cytokine storms, and is also involved in viral replication.^107^ Inhibitors of the PI3K/Akt/mTOR pathway can diminish excessive inflammatory reactions, and thus could ameliorate the course of COVID‐19.^107^, ^179^ Studies have shown that the PI3Kδ inhibitor idelalisib, alone or together with the antihistaminic ebastine, suppress the release of proinflammatory cytokines, such as IL‐1β, IL‐6, and TNF‐α. Accordingly, a clinical trial investigating the use of ebastine in association with an antiviral agent in the treatment of COVID‐19‐positive patients is underway (ChiCTR2000030535).^178^ AKT inhibitors, triciribine and MK‐2206, were reported to increase Tregs number and function, favoring lung injury recovery in endotoxin‐induced experimental lung injury mouse models. Therefore, blocking of Akt is proposed to increase patients Tregs in the lungs of patients with SARS‐CoV‐2 infection, thus diminishing the cytokines production and inflammation and promoting injury resolution.^180^ However, further experimental validation is needed in a suitable preclinical COVID‐19 model before clinical trials are able to commence for COVID‐19 patients. The critical role of mTOR in immune responses, cytokine storm, and Treg growth and activity suggests that mTOR inhibitors might exert beneficial effects against hyper‐reactivity during the critical phase of COVID‐19.^177^ Repurposed mTOR inhibitor (sirolimus, everolimus, temsirolimus, and tacrolimus) have been under preclinical and clinical investigation recently for severe influenza A/H1N1 pneumonia and acute respiratory failure. Further studies on mTOR inhibitors for therapeutic effects on COVID‐19 are well merited.^181^

The deregulation of inflammatory responses linked to NF‐κB and p38 MAPK was demonstrated for SARS‐CoV‐1, which leads to serious disease.^112^ Subsequently, the synthesis of cytokines, including IL‐6, TNF‐α, and β, is boosted after the activation of the NF‐κB and MAPK signaling pathway.^128^ Therefore, repurposing of inhibitors targeting NF‐κB and MAPK signaling is proposed as a COVID‐19 therapy. Several reported anti‐inflammatory or antiviral drugs for COVID‐19, such as HCQ, macrolide antibiotics, dexamethasone, and N‐acetylcysteine, are related to NF‐κB inhibition.^183^, ^184^, ^185^, ^186^ There are other reported inhibitors targeting the NF‐κB pathway, such as the anti‐inflammatory agents, phillyrin (KD‐1) and pyrazole derivative, which significantly reduced replication of SARS‐CoV‐2 and expression of proinflammatory factors.^322^, ^323^ Therapeutic inhibition of p38 has also been shown to attenuate COVID‐19 infection and inflammation. A preclinical study showed that inhibition of p38 with SB203580, a highly specific inhibitor of p38 MAPK, impaired SARS‐CoV infection in a mouse model.^182^, ^187^ There are p38 inhibitors, such as losmapimod (NCT04511819), HCQ (NCT04340544, NCT04342221), and silymarin (NCT04394208), which are in the clinical stages of development and were considered for treatment of severe COVID‐19 cases. However, most of these trials were eventually terminated, suggesting further efforts are needed to investigate the efficacy of highly selective and potent MAPK inhibitors for COVID‐19.

The JAK/STAT pathway is the downstream of IL‐6 and other cytokines involved in cytokine storm during viral infection.^132^ Therefore, JAK/STAT inhibitors are potentially valuable therapeutic options for COVID‐induced cytokine storm. Several JAK inhibitors, including tofacitinib, which targets JAK1 and JAK3, and baricitinib and ruxolitinib, both which target JAK1 and JAK2, have been under clinical investigation for treatment of SARS‐CoV‐2.^130^, ^188^, ^189^ Tofacitinib was reported to show mortality and pneumonia benefit in patients with COVID‐19, according to clinicaltrials.gov. There are several clinical trials underway for evaluating the therapeutic effects of tofacitinib in COVID‐19 patients (NCT04390061, NCT04332042, NCT04412252, NCT04415151, NCT04469114, NCT04750317). Treatment with baricitinib significantly improved the inflammatory condition associated with SARS‐CoV‐2‐infection and also limited lung pathology. There are a series of clinical trials evaluating baricitinib as a therapeutic for COVID‐19. One observation was robust reduction of serum IL‐6, IL‐1β, and TNF‐α and an increase in T and B cell counts, and increased production of antibodies against SARS‐CoV‐2 (NCT04438629). Another observation was that baricitinib protected lung function in patients with moderate‐to‐severe COVID‐19.^190^ There are numerous clinical trials investigating baricitinib as a therapeutic for severe COVID‐19. Despite these promising clinical data, the side effects of JAK inhibitors, such as increased risk of secondary infection and elevated risk of thrombosis, need to be addressed before further trials can be carried out (US FDA Drug Safety Communication).

GSK‐3 also incites systemic inflammation, which worsens disease through synthesizing the proinflammatory factors like IL‐6, IL‐1β,IL‐18, IFN‐γ, and TNF‐α.^136^ GSK‐3 inhibitors were reported to be effective in treating type 2 diabetes or neurodegenerative and psychiatric disorders; they are now being considered as novel therapeutic agents for COVID‐19.^191^, ^192^, ^193^, ^194^ Three GSK‐3 inhibitors, LY2090314, and AZD‐1080 and COB‐187, were observed to attenuate Spike protein‐induced chemokine CXCL10 expression in SARS‐CoV‐2‐infected human macrophage cells,^195^ providing justification for further exploration of GSK‐3 inhibitors as potential therapeutics for severe COVID‐19 (Figure 4).

#### 3.3.3 Repurposed potential therapies

Mesenchymal stem cells (MSCs) are an effective immunotherapy for the treatment of several disorders, including graft‐versus‐host disease, inflammatory bowel disease, osteoarthritis, RA, and multiple sclerosis. Recently, much attention has been paid to the use of MSCs for reducing pathological changes in the lungs.^324^ MSC‐based therapy is an option for SARS‐CoV‐2 infection, because of the immunomodulatory, anti‐inflammatory, and regenerative properties. Moreover, MSC therapy was found to be safe in preclinical and clinical studies.^220^ There are presently over 80 clinical trials registered at ClinicalTrials.gov for the treatment of COVID‐19.^220^

Another option for severe COVID‐19, for control of inflammatory cytokines and coagulopathy, is blood purification therapy, which was previously used for various severe refractory disorders, such as fulminant liver failure, transplant rejection and collagen diseases.^219^ Clinical studies have revealed beneficial clinical effects associated with therapeutic plasmapheresis. There are four blood purification technologies: (1) therapeutic plasma exchange (TPE), (2) AN69 (Oxiris) surface‐treated membranes, (3) Cytosorb, and (4) polymyxin b hemoperfusion.^325^

TPE effectively separates plasma from blood cells and replaces it with fresh frozen plasma. Case reports have been published showing successful TPE treatment for patients with severe COVID‐19.^326^, ^327^, ^328^ Numerous clinical studies have been carried out to evaluate the therapeutic effect of TPE on severe COVID‐19 cases, supporting the efficacy of TPE.^329^, ^330^, ^331^ Oxiris is a newly developed CKRT hemofilter with an AN69 surface treatment membrane that offers high endotoxin absorption and excellent antithrombotic properties based on negatively–positively charged attraction. The effectiveness of this approach has been reported, namely in reducing cytokine levels in patients with severe COVID‐19 disease.^332^, ^333^, ^334^ Cytosorb is a hemadsorption device that lowers cytokine levels associated with various critical diseases, such as septic shock and cardiac surgery; Cytosorb was approved in the European Union in 2011^335^, ^336^ and is currently being repurposed for treatment of pneumonia associated with severe COVID‐19.^337^, ^338^ Cotreatment with Cytosorb and venous–venous extracorporeal membrane oxygenation (ECMO) has been investigated in several clinical studies and has shown therapeutic benefit by decreasing cytokines and symptoms of ARDS (NCT04518969, DRKS00021447, NCT04344080). Polymyxin b hemoperfusion (PMX‐DHP) is a widely used blood purification therapy, now considered for treatment of severe COVID‐19, which decreases circulating endotoxins through adsorption to polymyxin b‐immobilized columns for septic shock patients.^339^ PMX‐DHP has been shown to decrease levels of IL‐6 and other inflammatory chemokines, however efficacy has been questionable.^340^, ^341^, ^342^

## 4 REPURPOSED DRUGS IN CLINICAL TRIALS

Pharmaceutical companies globally are focusing on developing medicines against SARS‐CoV‐2. However, the initial investment in new medical research and development has been expensive, the process is lengthy, and clinical trial outcomes are uncertain. Therefore, it is attractive to repurpose currently available drugs that target key proteins important for COVID‐19. Currently, many types of drugs, including antiviral agents, antibiotics, kinase inhibitors, and immunoregulators, are being investigated in clinical trials as monotherapy or as part of combination therapy for the treatment or prevention of COVID‐19 (Table 3).^249^, ^263^, ^343^, ^344^

**TABLE 3:** Summary of repurposed drugs that are approved or in clinical trials.

Drugs	Pathogenic Targets	Original indications	Clinical trails	References
Chloroquine (CQ) and hydroxychloroquine (HCQ)	Interference in endocytic pathway, blockade of sialic acid receptors, restrict spike protein cleavage, and prevent cytokine storm	Malaria and rheumatoid arthritis	Phase II, III, or IV NCT04328493, NCT04481633, NCT04333225, NCT04466540, and so on Single dose or different combinations (the effects were unsatisfactory)	^343^, ^345^, ^346^, ^347^, ^348^, ^349^
Azithromycin	Interference ligand/CD147 receptor interactions and anti‐inflammatory properties	Treatment of bacterial infections	Phase II or III NCT04332107, NCT04622891, NCT04622891, and so on Single dose or different combinations (the effects were unsatisfactory)	^350^, ^351^, ^352^, ^353^, ^354^, ^355^
Lopinavir	The viral proteases, 3CLpro and ACE2	HIV	Phase IV NCT04307693, NCT04738045, and so on Different combinations with ritonavir for pneumonia associated with COVID‐19 (the effects were unsatisfactory)	^242^, ^344^, ^356^, ^357^, ^358^
Cepharanthine	Prevent Sp‐ACE2 binding, act as an entry inhibitor in a prophylactic role	Agranulocytosis caused by tumor chemotherapy and radiotherapy and leukopenia caused by other causes	Phase II NCT05398705	^359^, ^360^
Bemcentinib	A highly selective and potent AXL inhibitor with antiviral activity	Advanced lung adenocarcinoma	Phase II trial in UK NCT04890509	^361^, ^362^, ^363^
Remdesivir (Veklury)	RdRp	Ebola	US FDA approved in May 2020 NCT04431453, NCT04365725, and so on	^364^, ^365^, ^366^, ^367^
Azvudin (Jie Beian, Henan Zhenzhen Biotechnology)	RdRp inhibitor, nucleoside‐based antiviral agents: nucleoside analogs mimic natural nucleosides	HIV, HBV, and HCV	NDA approved in July 2022 NCT04668235, NCT05033145, and so on	^368^
Favipiravir (Avigan, Zhejiang Hisun Pharmaceutical)	RdRp	Treatment of influenza virus infection	US FDA approved in 2020 Phase II or III NCT04464408, NCT04358549, NCT04351295, and so on	^369^, ^370^, ^371^
Molnupiravir (Lagevrio, Merck)	RdRp	Treatment of influenza virus infection	UK approved in Nov. 2021, FDA (EUA) and PMDA (Japan) approved, NDA (submitted) NCT04575597, NCT05595824, and so on	^372^, ^373^, ^374^
VV116 (JT001, Junshi Biosciences)	RdRp	COVID19	US FDA approved in Dec. 2021 NCT05227768, NCT05201690, NCT05221138	^18^, ^375^
PF‐07321332, (Paxlovid, Pfizer)	Mpro	COVID19	US FDA approved in Dec. 2021 NCT04756531, NCT04909853, NCT05011513, and so on	^376^
Ensitrelvir (Xocova, Shionogi)	3CLpro	COVID19	Phase II or III NCT05041907 JRCT2031210350 Approved (ERAS) in Japan in November 2022	^377^, ^378^
Masitinib (Masivet, AB Science)	3CLpro	Gastrointestinal stromal tumor	Phase II NCT05047783, NCT04622865	^379^, ^380^, ^381^, ^382^
Sabizabulin	Microtubule disruptor that has dual antiviral and anti‐inflammatory activities (cytokine Storm)	COVID19	FDA approved (EUA) in May 2022 NCT04842747 (the effects were dubious)	^383^, ^384^
Baricitinib (Olumiant, Eli Lilly Nederland)	Immune modulator, an inhibitor of JAK	Rheumatoid arthritis, alopecia areata	FDA approved (EUA) in November 2020, in July 2021 broadens existing emergency use, PMDA (Japan), approved in 2021 NCT05082714, NCT04970719, and so on	^263^, ^385^, ^386^
Anakinra (Kineret, Swedish Orphan Biovitrum)	Recombinant human IL‐1 receptor antagonist, management of cytokine Storm, severe respiratory failure and virus infection	Autoinflammatory diseases	Phase II or III NCT04680949, NCT04443881, NCT04643678, and so on	^145^, ^146^, ^147^, ^148^, ^239^, ^387^
Tocilizumab (RoActemra, Roche Regestration)	Recombinant humanized monoclonal antibody targeting the interleukin‐6 receptor (IL‐6R)	Castleman's disease, rheumatoid arthritis, giant cell arteritis, systemic juvenile idiopathic arthritis, cytokine release syndrome induced by CAR‐T therapy, polyarticular juvenile idiopathic arthritis	NDA approved in March 2020, and new clinical trials in Phase II, III, or IV NCT04445272, NCT04730323, NCT04331795, and so on	^140^, ^142^, ^143^, ^144^, ^388^

Data sources: https://clinicaltrials.gov.

### 4.1 CQ and HCQ

CQ is a Toll receptor and autophagy inhibitor.^345^, ^346^, ^347^ It is widely used to treat malaria and RA. In vitro, CQ was found to be effective in inhibiting COVID‐19 infection.^343^ Mechanistic studies have shown that CQ can target multiple key proteins of the SARS‐CoV‐2 virus,^348^, ^349^ such as the ACE2 and Mpro.^227^, ^238^ Currently, CQ is being investigated in numerous clinical trials for COVID‐19, including for medical staff as a preventative agent for COVID‐19, and measurement of its therapeutic effect across different stages of SARS‐CoV‐2 infection. Furthermore, various combinations of CQ and other drugs are being investigated in clinical trials, with both encouraging as well as controversial effects in patients.^351^, ^390^

### 4.2 Azithromycin

Azithromycin is an antibiotic that is widely used in the study and treatment of bacterial infections.^350^, ^351^, ^352^ It was discovered to be able to target many key COVID‐19 proteins.^353^ Current clinical trials are investigating the combination of azithromycin with other drugs against COVID‐19, including clarithromycin (NCT04622891), CQ (NCT04358068), and zinc preparations (NCT04370782). Azithromycin may cause aberrant alterations in the electrical activity of the heart, as well as potentially fatal irregular heartbeats.^354^ This risk is particularly severe in people with specific medical conditions, including patients with low potassium levels and those with allergies to antibiotics, as well as in those patients using drugs to treat irregular cardiac rhythms. In some COVID‐19 trials, azithromycin failed to accomplish the specified end point.^355^

### 4.3 Lopinavir

Lopinavir is an AIDS therapeutic drug that selectively inhibits HIV‐1 protease, inhibits HIV‐1 maturation, and prevents HIV infectivity.^356^, ^357^ It was discovered that lopinavir inhibits the activity of 3CLpro and PLpro in SARS‐CoV‐2.^249^, ^344^ Lopinavir in combination with different antiviral drugs has been investigated in clinical trials for the prevention and treatment of COVID‐19. Although lopinavir did not improve outcomes for people with mild COVID‐19 compared with standard care, lopinavir–ritonavir may be associated with a significant reduction in total mortality.^242^, ^358^

### 4.4 Cepharanthine

Cepharanthine is an extract of Stephania chinensis, a traditional Chinese medicine, which was traditionally used to increase the amount of white blood cells and boost the body's immunity to tumors; it can also be used to treat silicosis and hair loss.^359^, ^360^ Through inhibiting NF‐κB activation, NO production, lipid peroxidation, cyclooxygenase expression, and cytokine production, cepharanthine exhibits activity against viral replication and inflammatory responses.^360^ Cepharanthine was recently discovered in preclinical models to be the most potent coronavirus inhibitor out of 2406 clinically licensed drug repurposed candidates (NCT05398705). Cepharanthine has entered clinical trials and subsequent market use, providing a new and promising option for COVID‐19 prevention and control.

### 4.5 Bemcentinib

Bemcentinib is an efficient and selective AXL inhibitor.^361^ The US FDA‐approved bemcentinib and the PD‐1 antibody, pembrolizumab, as fast‐track designation for the treatment of advanced patients with STK11 mutations and patients with metastatic non‐small cell lung cancer. The antiviral potential of bemcentinib against the Ebola and Zika viruses has been confirmed and validated.^362^, ^363^ Bemcentinib is now being studied in a clinical trial for the treatment of COVID‐19. The major goal of the study is to assess the efficacy of bemcentinib as an add‐on to standard of care treatments in hospitalized patients with COVID‐19 (NCT04890509). The off‐target effect of kinase inhibitors remains a concern in patients not diagnosed with cancer, and bypass activation can also cause patients to develop drug resistance to kinase therapies. However, the anti‐SARS‐CoV‐2 activity of kinase inhibitors continues to be worth researching considering the host of potential therapeutic benefits associated with these agents.

### 4.6 Favipiravir (Avigan)

Favipiravir is an analog of guanine and an effective and selective inhibitor of RNA polymerase; it is approved in Japan for the alternative treatment of influenza.^369^, ^370^ In vitro, nucleoside analogs have various antiviral mechanisms of action, including lethal mutation, strand termination, and inhibition of nucleotide biosynthesis.^371^ There are currently over 30 clinical trials investigating the efficacy of favipiravir as a single agent or as part of combination therapy. Favipiravir was approved as a repurposed agent for the management of COVID‐19 in 2020. However, because nsp14‐ExoN was discovered to have an RNA proofreading activity, SARS‐CoV‐2 gained nucleoside analog resistance.^391^, ^392^ Ribavirin has relatively little antiviral impact on coronaviruses in vitro, and similarly, favipiravir has a limited effect on coronaviruses.

### 4.7 Molnupiravir (Lagevrio)

Molnupiravir is an oral prodrug for the ribonucleoside analog EIDD‐1931. Molnupiravir has exhibited substantial activity against influenza viruses and coronaviruses, and therefore it has been investigated in several clinical trials due to its therapeutic potential as a therapy for COVID‐19 and seasonal treatment.^372^, ^373^, ^374^ A phase III clinical trial examined the efficacy of molnupiravir in nonhospitalized high‐risk adult patients with mild‐to‐moderate COVID‐19. The findings of this trial imply that starting molnupiravir early in high‐risk adult patients without COVID‐19 immunization reduced the risk of hospitalization or death significantly (NCT04575584). On December 23, 2021, the US FDA approved an Emergency Use Authorization (EUA) for molnupiravir for the treatment of COVID‐19 in adult patients with mild to moderate coronavirus disease.

### 4.8 VV116 (JT001)

VV116 (JT001), an oral nucleoside analog antiviral drug, that is one of the most focused China‐developed COVID‐19 treatment candidates.^18^ Metabolized from VV116, the parent nucleoside (116‐N1) is intracellularly converted to the nucleoside triphosphate active form, which would interfere with the function of RdRp of SARS‐CoV‐2, thus exerting antiviral effects.^375^ There are many clinical trials (NCT05227768, NCT05201690, NCT05221138) that have been accomplished at the Phase I Clinical Research Center of Shanghai Xuhui Central Hospital, which showed satisfactory effects on safety, tolerance, and pharmacokinetics characteristics. On December 31, 2021, the EUA of VV116 has been approved for the treatment of COVID‐19 in Uzbekistan. And on January 29, 2023, VV116 was approved for listing with conditions in the State Food and Drug Administration of China (BioSpace news).

### 4.9 Remdesivir (Veklury)

Remdesivir, a nucleoside analog with antiviral activity, was originally created as an Ebola treatment by inhibiting RdRp activity, however clinical results were negative.^364^, ^365^ The first case of a COVID‐19 patient successfully treated with remdesivir in the United States prompted research into remdesivir for COVID19. Further research has indicated that remdesivir potentially inhibits COVID‐19 activity through acting on the RdRp of COVID‐19.^366^ Remdesivir was approved by the US FDA for COVID‐19, and there are currently 60 ongoing clinical trials investigating its efficacy. Antiviral nucleic acid analogs, such as ribavirin, are used to treat coronavirus infections and will be cut out by the coronavirus exoribonuclease ExoN when incorporated into the viral RNA. However, because remdesivir is resistant to ExoN, it is more successful in treating coronavirus than other nucleic acid agents.^391^, ^392^

### 4.10 Azvudine (Jie Beian)

Azfudine is the world's first dual‐targeting inhibitor that targets HIV reverse transcriptase with the accessory protein Vif. This agent has exhibited antiviral activity against HIV, HBV, and HCV and inhibits NRTI‐resistant viral strains, as well as HIV‐1 and HIV‐2.^368^ On July 20, 2021, the CDE conditionally approved the combination of this product with other reverse transcriptase inhibitors for adult HIV‐1‐infected patients with high viral load. Given its excellent antiviral effects, azfudine is under clinical investigation for the treatment of COVID‐19 as a single agent. On July 25, 2022, an application was filed for azfudine as a treatment for SARS‐CoV‐2‐associated pneumonia by the State of Food and Drug Administration of China.

### 4.11 PF‐07321332, PAXLOVID™ (PF‐07321332 and Ritonavir)

PF‐07321332 is a highly effective, orally active 3CLPRO inhibitor for SARS‐CoV‐2. It is a trypsin‐like protease inhibitor that inhibits sodium channel function in airway epithelial cells.^376^ On December 22, 2021, the US FDA approved the first oral drug, Paxlovid, to be used as an emergency treatment for SARS‐CoV‐2 infection. Paxlovid was investigated as a treatment for children and adults with SARS‐CoV‐2 (mild to moderate COVID‐19 in adults and those aged 12 years old, with 40 kg body mass, and in a patient population with a higher risk of severe illness). Preliminary results of clinical trials showed an 89% lower risk of hospitalization and death from any cause in patients treated within 3 days of developing symptoms, as compared with placebo.

### 4.12 Ensitrelvir (Xocova)

Ensitrelvir is the first orally active, noncovalent, nonpeptide 3CL protease inhibitor developed for SARS‐CoV‐2.^377^, ^378^ Phase II clinical data showed that SARS‐CoV‐2 viral load decreased by approximately 63–80% in asymptomatic and mildly symptomatic patients after four days, compared with placebo, and was also effective against the Omicron strain. In addition, there are more than a dozen clinical studies being conducted around ensitrelvir according to ClinicalTrials.gov. On November 22, 2022, ensitrelvir was approved in Japan for the treatment of COVID‐19 under the Emergency Regulatory Approval System (New Atlas). Although efficacy was demonstrated, ensitrelvir is slightly inferior to Pfizer's Paxlovid. Ensitrelvir has a half‐life of 10−30 h and has been demonstrated to work in the absence of ritonavir in vitro, improving its performance in safety and patient compliance.

### 4.13 Masitinib (Masivet)

Masitinib is a potent, orally available, and selective c‐Kit inhibitor that also inhibits PDGFRα/β, Lyn, Lck, FGFR3, and FAK.^379^ Masitinib exhibits potential antiproliferative and proapoptotic activity, with low toxicity.^380^ It plays an important role in the mast cell activation process, such as the immune response, lymphatic infiltration in the brain, and the inflammatory response associated with multiple sclerosis.^381^ Masitinib is mainly used in the treatment of gastrointestinal stromal tumors caused by the c‐KIT mutation (gain of function) (NCT00812240). Studies have shown that masitinib can competitively inhibit the activity of the main protease, 3CLpro, thus inhibiting the replication of SARS‐CoV‐2. Currently, there are two clinical trials that are underway for evaluating the efficacy in mono‐ or combination treatments, according to ClinicalTrials.gov.

### 4.14 Sabizabulin

Sabizabulin is a highly efficient and orally available α/β tubulin (tubulin) inhibitor, which can destroy the intracellular transport of the virus along microtubules. With strong antiviral activity and a demonstrated anti‐inflammatory effect, this agent was believed to have the potential to alleviate cytokine storm caused by the novel coronavirus.^383^, ^384^ Despite these promising early findings, sabizabulin subsequently failed the expert advisory committee's examination, and clinical trials were terminated due to efficacy.

### 4.15 Baricitinib (Olumiant)

Baricitinib (Olumiant) is a dual JAK1 and JAK2 inhibitor that was first approved for therapy of adults with severe alopecia areata.^393^ Baricitinib is also a prescription medicine used to treat adults with moderately to severely active RA.^394^ Recently, baricitinib has been repurposed for treatment of COVID‐19.^263^ Baricitinib showed high efficacy against SARS‐CoV‐2 migration and intracellular assembly and was effective in treating ARDS in patients with COVID‐19.^263^ A clinical trial investigating baricitinib plus remdesivir was associated with fewer serious adverse events and superior to remdesivir alone in reducing the recovery time among patients with COVID‐19. This was notable among those patients requiring supplemental oxygen, noninvasive, or invasive mechanical ventilation (NCT04401579). On November 19, 2020, the US FDA approved an EUA for baricitinib in combination with remdesivir for the treatment of COVID‐19 in hospitalized adults and pediatric patients receiving high‐flow oxygen or noninvasive ventilation or ECMO.^385^ As the first approved COVID‐19 immunomodulatory treatment agent, baricitinib was authorized as a stand‐alone treatment on July 28, 2021.^386^

### 4.16 Anakinra (Kineret)

The IL‐1 receptor antagonist, anakinra (Kineret), was first approved for the treatment of RA in 2001 and is now being used as a therapy for COVID‐19.^145^, ^146^, ^147^, ^148^ Some clinical studies suggest that anakinra may improve the prognosis of patients with moderate to severe COVID‐19 through reducing the mortality risk in patients with pneumonia and hyperinflammation.^387^ Therefore, anakinra may be a safe, anti‐inflammatory treatment option to combat COVID‐19. Currently, there are over 30 clinical trials underway for evaluating the efficacy in mono‐ or combination treatments, according to the ClinicalTrials.gov.^148^, ^149^ On December 16, 2021, the European Medicines Agency authorized extension of the use of anakinra across the EU to treat COVID‐19 in adult patients with pneumonia who need oxygen supplementation and who are at a high risk of developing severe respiratory failure.

### 4.17 Tocilizumab (RoActemra)

The IL6R antibody, tocilizumab, previously approved for RA and used in the treatment of cytokine storm caused by chimeric antigen receptor T cell (CART) therapy,^143^ is now under consideration as a COVID‐19 therapy. Through blocking cytokine production, ameliorating the symptoms of systemic toxicity, and reducing the requirements for adjuvant and other therapies, this antibody has shown high efficacy and safety in a series of preclinical and clinical studies involving SARS‐CoV‐2.^140^, ^142^, ^144^, ^388^ Up to now, there have been more than 30 SARS‐CoV‐2 trials (NCT04445272, NCT04730323, NCT04331795, so on) investigating tocilizumab as a COVID‐19 therapeutic. On June 24, 2021, tocilizumab was approved by the US FDA for the treatment of hospitalized adults and pediatric patients (over two years old) with severe symptoms of COVID‐19 (FDA News Release).

## 5 DISCUSSION AND FUTURE PROSPECTS

The outbreak caused by SARS‐CoV‐2 threatened the world with increasing infection and massive casualties.^1^, ^2^ During the pandemic, the SARS‐CoV‐2 subvariants evolved rapidly with enhanced transmissibility and the alarmingly rising rate of infection. The constantly mutated S protein under high‐pressure selection rendered vaccines and antibodies somewhat less effective. An inhaled version of the COVID‐19 vaccine has been shown to elicit potent immune responses against different subvariants of concern, including the Omicron variant^395^, ^396^, ^397^; China has approved the world's first inhaled COVID‐19 vaccine (September, 2022) for use as a booster (Euronews, C&EN). Despite this, reinfections are still common. It is anticipated that the SARS‐CoV‐2 virus showing reduced mortality but will persist for an extended length of time, and studies have shown that immunosuppressed individuals are unable to mount responses even after two vaccine doses.^8^, ^9^ Thus, other options are needed to protect against SARS‐CoV‐2.

There is an urgent need to identify potential pathogenic proteins or pathways that are highly conserved among multiple virus, such as Ebola virus, MERS‐CoV, and more recently, coronaviruses.^11^, ^12^, ^13^ There are two main categories of pathogenesis and druggable molecular targets that are involved in SARS‐CoV‐2 infection and symptoms, including (1) the viral targets associated with SARS‐CoV‐2 and (2) the host cell protein targets that are essential for viral life or virus–host cell response^14^, ^20^, ^21^, ^22^, ^23^, ^24^ (Figure 1). Key potential targets associated with coronaviruses, such as the replication‐related enzymes RdRp, protease Mpro/3Cpro and PLpro, and the host‐cell receptors ACE2, AXL, and CD147, are conserved among coronaviruses.^11^, ^12^, ^13^, ^30^, ^44^, ^47^ Host cell responses to SARS‐CoV‐2 are associated with the development of symptoms of COVID‐19. There are many potential targets that play critical roles in this process and that could be exploited therapeutically. For example, proteins such as EGFR, ABL, SFKs, and CDKs are involved in various pathologies associated with SARS‐CoV‐2 infection, including pneumonia, fibrosis, ARDS, and cytokine storm.^77^, ^78^, ^79^, ^85^, ^97^, ^98^, ^114^, ^115^, ^116^ The complex immunopathological manifestations of COVID‐19 are associated with many signaling pathways, including IL‐6/IL‐6R, JAK/STAT signaling, NF‐κB, and MAPK pathways, which induce the expression of proinflammatory cytokines and hyperinflammation, and many therapies have been developed accordingly^128^, ^130^, ^138^, ^139^, ^188^, ^189^ (Table 1). There are ongoing efforts to repurpose drugs that can effectively inhibit potential therapeutic targets and thus suppress the transmission of SARSCoV2 or ameliorate the symptoms of COVID19.

Cost‐effective and timeline‐reducing drug repurposing approaches could be a viable treatment alternative for COVID‐19 to the more traditional drug development process.^14^ Various strategies are employed to find alternative uses of an approved or investigational drug outside of its original indications, including computational approaches that using algorithms to study drug interactions and model disease, and experimental, laboratory‐based assays and screening of drug libraries.^11^, ^14^, ^15^, ^30^, ^227^, ^245^, ^249^ There are numerous successful drug repurposing assays performed for reported or repurposed pathological targets that lead to preclinical or clinical evaluation of identified candidates for potential therapeutic activity against COVID‐19 (Table 2).

There are many promising alternative drugs that are being investigated in clinical trials or that have been approved for treating COVID‐19 patients, either alone or in combination with other treatments (Table 3). Some repurposed drugs target the viral protein RdRP, such as remdesivir,^364^, ^365^, ^366^, ^367^ favipiravir,^369^, ^370^, ^371^ and molnupiravir,^372^, ^373^, ^374^ and have been approved and provided benefit for moderate‐to‐severe cases of COVID‐19. In addition, numerous kinase inhibitors used to treat conditions such as chronic inflammations or autoimmune diseases have been found to also exhibit antiviral activity, anticytokine storm activity, and antifibrotic activity due to selective targeting of important virus‐associated proteins. Examples include the mTOR inhibitor rapamycin and tacrolimus,^179^, ^273^ the JAK2 inhibitor fedratinib,^292^ the PI3Kδ inhibitor idelalisib and ebastine,^178^ and the JAK2–STAT inhibitors, baricitinib and tofacitinib.^263^, ^385^, ^386^ Several alterative inhibitors targeting kinases or cytokine receptors that have shown promising therapeutic activity in various clinical trials have recently been approved for patients. Examples are the JAK inhibitor baricitinib (Olumiant),^263^, ^385^, ^386^ recombinant human IL‐1 receptor antagonist anakinra (KINERET),^145^, ^146^, ^147^, ^148^, ^387^ and tocilizumab,^140^, ^142^, ^143^, ^144^, ^388^ which is a recombinant humanized monoclonal antibody targeting the IL‐6R (Table 3).

When repurposing authorized drugs for new therapeutic uses for COVID‐19 patients, the specificity of inhibitors on target proteins must be considered in order to avoid adverse effects/unwanted toxicity, and short‐term therapy could be investigated for mitigation of unwanted side effects as well. Combination therapy is a promising approach that could be employed for higher treatment efficacy. Antiviral drugs are often combined with other antiviral agents having different targets, such as ritonavir‐lopinavir,^242^, ^358^ and antiviral drugs are also combined with kinase inhibitors, such as remdesivir–baricitinib.^385^ In addition, kinase inhibitors are combined with compounds/antibodies with anti‐inflammatory or antifibrotic activity, for example, baricitinib–tocilizumab,^398^ the combination of which was found to be superior to monotherapy in reducing recovery time and accelerating improvement in clinical status. Successful outcomes for COVID‐19 patients with access to these drug combination approaches support the notion that the repurposing strategies can be beneficial for controlling outbreaks like SARS‐CoV‐2.

Global collaborative efforts between researchers and healthcare professionals toward drug discovery and development is continuously needed to prevent future pandemics and to restrict the spread of current problematic contagions. In addition to immunosuppressed individuals who are unable to mount responses even after two vaccine doses,^8^, ^9^ there are the elderly and people afflicted with underlying health conditions that make them more susceptible to COVID‐19, such as cancer, diabetes, cardiovascular disease, and other immune‐related diseases.^399^ So far, vaccines remain the best option for protection from infection. Continuing the vaccination program that has been launched globally and investing in development of novel and more effective vaccines or prophylactics against infection are a top priority. Identification of potential therapeutic drug targets using multidisciplinary approaches, such as genomic studies, structural biology studies, proteomic studies, chemoinformatic studies, and computational tools could help facilitate the development of effective and specific antiviral medicines against SARS‐CoV‐2 infection. In addition, combination of newly identified and repurposed therapeutics with the other agents or therapeutic approaches will further help toward controlling the spread of diseases like COVID‐19.

## AUTHOR CONTRIBUTIONS

Y. X., H. M., and J. Y. wrote the manuscript and prepared figures and tables. Y. C. helped with figures and tables. J. Y., E. W., Q. L., and J. G. provided valuable scientific feedback. All authors have read and approved the final manuscript.

## CONFLICT OF INTEREST STATEMENT

The authors declare no conflict of interest.

## ETHICS STATEMENT

Not applicable.

## Data Availability

Not applicable.
